# Sub-MIC antibiotics influence the microbiome, resistome and structure of riverine biofilm communities

**DOI:** 10.3389/fmicb.2023.1194952

**Published:** 2023-08-01

**Authors:** Gabriela Flores-Vargas, Darren R. Korber, Jordyn Bergsveinson

**Affiliations:** ^1^Food and Bioproduct Sciences, University of Saskatchewan, Saskatoon, SK, Canada; ^2^Watershed Hydrology and Ecology Research Division, Environment and Climate Change Canada, Saskatoon, SK, Canada

**Keywords:** antibiotics, antibiotic resistance, biofilms, environmental resistome, microbiome, sub-MIC

## Abstract

The effects of sub-minimum inhibitory concentrations (sub-MICs) of antibiotics on aquatic environments is not yet fully understood. Here, we explore these effects by employing a replicated microcosm system fed with river water where biofilm communities were continuously exposed over an eight-week period to sub-MIC exposure (1/10, 1/50, and 1/100 MIC) to a mix of common antibiotics (ciprofloxacin, streptomycin, and oxytetracycline). Biofilms were examined using a structure–function approach entailing microscopy and metagenomic techniques, revealing details on the microbiome, resistome, virulome, and functional prediction. A comparison of three commonly used microbiome and resistome databases was also performed. Differences in biofilm architecture were observed between sub-MIC antibiotic treatments, with an overall reduction of extracellular polymeric substances and autotroph (algal and cyanobacteria) and protozoan biomass, particularly at the 1/10 sub-MIC condition. While metagenomic analyses demonstrated that microbial diversity was lowest at the sub-MIC 1/10 antibiotic treatment, resistome diversity was highest at sub-MIC 1/50. This study also notes the importance of benchmarking analysis tools and careful selection of reference databases, given the disparity in detected antimicrobial resistance genes (ARGs) identity and abundance across methods. Ultimately, the most detected ARGs in sub-MICs exposed biofilms were those that conferred resistance to aminoglycosides, tetracyclines, β-lactams, sulfonamides, and trimethoprim. Co-occurrence of microbiome and resistome features consistently showed a relationship between Proteobacteria genera and aminoglycoside ARGs. Our results support the hypothesis that constant exposure to sub-MICs antibiotics facilitate the transmission and promote prevalence of antibiotic resistance in riverine biofilms communities, and additionally shift overall microbial community metabolic function.

## 1. Introduction

Over the last decade, many studies have examined anthropogenic systems with high rates of antibiotic resistance events, colloquially-known as “Antimicrobial resistance (AMR) hotspots” (Karkman et al., 2018; Kraemer et al., 2019; Kunhikannan et al., 2021; Yadav and Kapley, 2021). Monitoring these hotspots (i.e., clinical settings, wastewater treatment plants, and pharmaceutical manufacturing plants) is critical to understand mechanisms of acquired resistance and the prevalence, maintenance, and transmission risk of pathogenic bacteria. The linkage between AMR hotspots and increased dissemination of antimicrobial resistance genes (ARGs) and bacteria (ARB) is clear (Munk et al., 2022); however, relatively little is known of AMR abundance and dynamics downstream of hotspots where sub-minimum inhibitory concentration (sub-MIC) of antibiotics may still exert selective pressure on environmental microbiomes (Ebmeyer et al., 2021).

Exposure to sub-MICs of antibiotics have been shown to promote the upregulation of mutagenesis and DNA transfer events which can lead organisms within a mixed community to acquire ARGs (Chow et al., 2021). There is evidence that sub-MICs of antibiotics induce the expression of genes coding for virulence factors (Laureti et al., 2013) which can proliferate via horizontal gene transfer (HGT) mechanisms. HGT events themselves, along with associated integron and transposon recombination, have been shown to be induced or mediated by the bacterial SOS response (Baharoglu et al., 2010). Indeed, sub-MICs levels of aminoglycosides, fluoroquinolones and β-lactams have reported to directly increase mutation and conjugation rates via the SOS response in *Escherichia coli*, *Staphylococcus aureus*, and *Vibrio cholerae* (Baharoglu et al., 2013; Andersson and Hughes, 2014).

To date, most studies examining sub-MICs effects on microorganism behavior have focused on single-species of pathogenic bacteria, or at best, the same family of bacteria (Davies et al., 2006; Laureti et al., 2013; Abe et al., 2020). Multispecies experimental data is scarce, but recently, studies have surveyed antibiotic concentrations in environmental microbiomes and their relation to AMR (Danner et al., 2019; Chow et al., 2021; Sanchez-Cid et al., 2022). In freshwater aquatic systems, which are the primary receiving environments of wastewater treatment plants (WWTP) discharge and agricultural run-off, biofilm communities are abundant. Biofilms are known to support the maintenance and dissemination of antibiotic resistance mechanisms, genes and a diversity of organisms (Abe et al., 2020). The physical nature of biofilm architecture and lifecycle confer considerable protection from surrounding chemical stressors because of the production of extracellular polymeric substances (EPS). This EPS matrix, and the close proximity of constituent microorganisms, allows for adaptive tolerance mechanisms to be supported as well as the inter-organism exchange of genetic material by various means (Flemming et al., 2022). Thus, naturally occurring aquatic biofilms could act as potential reservoirs and maintenance environments of AMR in a myriad of conditions (Balcázar et al., 2015; Guo et al., 2018; Flores-Vargas et al., 2021; Matviichuk et al., 2022). Studies examining biofilms response to high concentrations of antibiotics have often focused on microbial communities in close proximity to WWTP effluents (Aubertheau et al., 2017; Lépesová et al., 2018; Chonova et al., 2019; Maestre-Carballa et al., 2019; Petrovich et al., 2019; Matviichuk et al., 2022). However, a clear understanding of biofilm community response to constant, sub-MICs of antibiotics and their role in promoting or maintaining AMR in supposed “non-impacted” environments is still lacking (Cairns et al., 2018). Whole community metagenomic analysis provides for necessary data to understand the transfer and prevalence of AMR in the environment, which are fundamental dynamics aimed to perform future environmental risk assessments. To determine the potential risk that low antibiotic concentrations present in aquatic biofilm systems, the range of antibiotic concentrations that have an effect in shaping naturally occurring microbial communities must be defined, as must the response of the communities’ tendency to the disseminate ARGs and ARB. Herein we characterize the response of natural riverine biofilm communities under selective presence of various sub-MIC antibiotics using metagenomic sequencing and biofilm architecture analysis, to determine whether exposure influences both community structure and function, and the abundance of ARGs and AMR-related functions.

## 2. Materials and methods

### 2.1. Experimental design

Microcosm experiments for biofilm development were performed in rotating annular bioreactor (RAB) systems which have been previously described in detail (Lawrence et al., 2000, 2004). Natural river water from the South Saskatchewan River (Saskatoon, Canada) was collected weekly and used as inoculum to establish the microbial biofilms, and as a source of carbon and nutrients for biofilm growth and development. Physicochemical parameters of the South Saskatchewan River were previously described (Lawrence et al., 2007a); where water was collected within city limits, yet upstream of both the municipal WWTP facility and sources of agricultural discharges. River biofilms were provided a one-week establishment phase without antibiotics to initiate growth on the surface of the 12 removable polycarbonate strips (1 × 11 cm) within each RAB. The 12 RABs were run in parallel, and each treatment was replicated (*n* = 3). RAB dose treatments were provided by the direct and constant addition of an antibiotic cocktail of three commonly used antibiotics from different drug classes at sub-MIC dosages of 1/10, 1/50, and 1/100 their respective MIC level, resulting in μg/L levels within the RAB system. Antibiotics concentration and degradation within the RAB system were not assessed after their addition.

MIC values for the biofilm communities were determined based on breakpoint reports of EUCAST (European Committee on Antimicrobial Testing EUCAST) and CLSI (Clinical Laboratory Standards Institute; CLSI, 2018; EUCAST, 2019): ciprofloxacin (MIC 0.5 mg/L; 1/10: 50 μg/L, 1/50: 10 μg/L, 1/100: 5 μg/L), streptomycin (MIC 512 mg/L 1/10: 51,200 μg/L, 1/50: 10,240 μg/L, 1/100: 5120 μg/L), and oxytetracycline (MIC 125 mg/L 1/10: 12,500 μg/L, 1/50: 2,500 μg/L, 1/100: 1,250 μg/L) were used in this study (Supplementary Table S1). The selection of these three antibiotic drug classes was based on the list of antimicrobial susceptibility surveillance criteria by the WHO, where streptomycin and ciprofloxacin are listed as critically important and oxytetracycline is considered as highly important (WHO, 2017). Additionally, ciprofloxacin is an antibiotic routinely found in WWTP discharge; and oxytetracycline and streptomycin are broad-spectrum antibiotics widely used in Canadian and global livestock operations (Kraemer et al., 2019).

Control RABs were operated with river water alone. Biofilms were grown under treatment and control conditions for 8 weeks following a one-week establishment period, after which strips were removed from each RAB, immediately frozen and stored at −80°C for subsequent molecular analyses. All analyses (see below) were conducted on subsamples from randomly selected biofilm strips from each RAB replicate.

### 2.2. Microscopic analysis

#### 2.2.1. Confocal laser scanning microscopy

Coupon pieces of 1 cm^2^ were excised from a randomly selected strip of each RAB and stained (below) prior to observation using a Confocal Laser Scanning Microscope Nikon Eclipse LV 110 DU and C2 camera with water-immersible lenses (10×, 40×, 60×; Nikon, Chiyoda, Tokyo, Japan). Biofilm architecture was quantified using a three-channel procedure (Neu et al., 2001; Dynes et al., 2006), where signals at green (excitation 488 nm, emission 522/32 nm), red (excitation 568 nm, emission 605/32 nm) and far-red channels (excitation 647 nm, emission 680/32 nm) were obtained. Coupons of 1 cm^2^ colonized by biofilm communities were directly stained with SYTO 9 (Molecular Probes, Eugene, OR, United States; excitation wavelength 488 nm, emission wavelength 522–532 nm) to detect nucleic acids of bacteria. EPS components of the biofilm matrix were visualized using three fluorescent fluor-conjugated lectin-binding dyes at 1 mg/mL: *Triticum vulgaris*-TRITC (TRITC: tetramethyl rhodamine isothiocyanate; excitation 568 nm, emission 605/32 nm; Sigma Chemicals, St. Louis, MI, United States) with polymer binding specificity for *N*-acetylglucosamine residues and oligomers; *Arachis hypogaea*-FITC (FITC: fluorescein isothiocyanate; excitation 485/495 nm, emission 510/600 nm; Sigma Chemicals, St. Louis, MI, United States) with polymer binding specificity for galactose and *N*-acetylglucosamine; and *Canavalia ensiformis*-FITC, also known as “Concanavalin A” (excitation 495/500 nm; emission 495/519 nm; Sigma Chemicals, St. Louis, MI, United States) with polymer binding specificity for mannose and glucose residues. Additionally, chlorophyl autofluorescence from algal and cyanobacteria cells (excitation 647 nm, emission 680/32 nm) was detected in the far-red channel (Lawrence et al., 2004; Dynes et al., 2006). Five areas per coupon were randomly selected for Z-stack image scanning with a slice interval of 5 μm (40×) and 1 μm (60×). CLSM image sequences were collected as previously described by Lawrence et al. (2005) and used for image analysis via ImageJ (Schneider et al., 2012) to define biofilm depth (or thickness) and architecture, and biomass of bacteria, EPS and autotrophs.

#### 2.2.2. Protozoa enumeration

Enumeration of protozoan organisms were also considered for overall biofilm community structure. Weekly counts were performed in water-immersed 1 cm^2^ coupons from each RAB using a light microscope and protozoa or micrometazoa were identified based on morphology and manually counted (Packroff et al., 2002).

### 2.3. Metagenomic analysis

#### 2.3.1. DNA extraction and whole genome sequencing

For each RAB, biofilm material from five randomly selected strips were recovered. Cellular biomass was aseptically obtained using a cell scraper (08-100-241; Fisher Scientific, Pittsburgh, PA) and the collected material centrifuged for 5 min at 9,000 × g to separate the water phase (Lawrence et al., 2009; Bergsveinson et al., 2020) and concentrate biofilm material to 300 mg wet weight. Total DNA was extracted using the Mag-Bind® Universal Pathogen Kit (Omega Bioservices, Norcross, GA, United States) and concentration and yield of DNA (5–10 ng/μL) was measured with the QuantiFluor dsDNA System on a Quantus Fluorometer (Promega, Madison, WI, United States). Whole genome libraries targeted for prokaryotic organisms were constructed using the KAPA Biosystems HyperPlus Kit following manufacturer’s instructions (Kapa Biosystems, Wilmington, MA, United States). Briefly, DNA was fragmented, ends were repaired, 3′ adenylated, and ligated to adapters. The resulting adapter-ligated libraries were PCR-amplified, Illumina indexes added, and pooled for multiplexed sequencing on an Illumina HiSeq4000/X10 platform (Illumina, San Diego, CA, United States) using the paired-end 150 bp run format, yielding 29 to 49 million reads per sample (average of 36 million reads; Supplementary Table S2).

#### 2.3.2. Data normalization

Paired-end raw reads were quality filtered with Trimmomatic v0.36 (Bolger et al., 2014) using the following parameters: HEADCROP:10 LEADING:3 TRAILING:3 SLIDINGWINDOW:4:15. Adapters were removed using the TruSeq3 adapter sequence file as reference. All samples were subsampled to 25 million reads (Mreads) using the seqkt tool package.^1^ Briefly, FASTQ reads depth from each sample was rarefied to the lowest number of Mreads/Sample, remaining reads were randomly discarded. This method ensured a uniform population diversity to perform comparative analyses (Connelly et al., 2017; Chekabab et al., 2020). Trimmed and subsampled FASTQ reads were then analyzed by alignment to different reference databases, as described below.

#### 2.3.3. Taxonomic profiling

Identification of bacteria and calculation of relative abundance at the genera level was assessed using three metagenome taxonomic profiling pipelines: CosmosID v2.0 (CosmosID Inc., Rockville, MD), Kraken v2 (Wood et al., 2019) and MetaPhlAn v3.0 (Segata et al., 2012). CosmosID is a web-based platform that utilizes data mining k-mer-based algorithms (Genius software) and Genbook, a high-performance curated comparator database, and includes references from NCBI-RefSeq, CARD, ARDB, VFDB, IMG and DDBJ and contains over 15,000 bacterial, 5,000 viral, 250 protists and 1,500 fungal species (Connelly et al., 2017; Junqueira et al., 2017; Yan et al., 2019; Chekabab et al., 2020). Taxonomic classification with the Kraken pipeline was performed with subsampled, trimmed FASTQ files using default parameters with the “—paired” option to indicate paired read files. Reads were mapped against the standard Kraken2 database comprised of complete genomes based on the NCBI RefSeq database (downloaded February 2022; O’Leary et al., 2016) for bacteria, viruses, fungi, protozoa and archaea. MetaPhlAn analysis was performed using the default parameters in addition to the “—ignore_eukaryotes*”* and “-t rel_ab_w_read_stats” options, the latter employed to estimate the number of reads per clade and obtain absolute abundance outputs for subsequent diversity indexes analysis. Microbial composition was compared against the CHOCOPhlAn_201901 database. Taxonomic abundance matrices from CosmosID, Kraken and MetaPhlAn analysis of each sample were prepared for analysis and visualization with all microbial taxa above 0.01% relative abundance included.

#### 2.3.4. Resistome analysis

The resistome profiles of ARGs from all samples were identified and compared through three different databases: CosmosID, the Comprehensive Antibiotic Resistance Database (CARD, v.3.0.0; Jia et al., 2016), and ResFinder v. 4.1.11 (Bortolaia et al., 2020). Metagenomes were first assembled using MEGAHIT v. 1.2.9 (Li et al., 2015) and contigs annotated with prokka v.1.14.5 (Seemann, 2014; Supplementary Table S2). Quality-assembled contigs were mapped against CARD and ResFinder databases. The RGI (Resistance Gene Identifier) application v 6.0.2 in *bwt* mode for metagenomic reads was used for CARD analysis. Analysis using CosmosID was performed by mapping the trimmed and concatenated paired reads (.fastq) against the platform’s database using default settings and using the filtering threshold to ensure high confidence. Output of CosmosID included abundance counts table estimates of ARGs downloaded for further data analysis. The virulome of each sample was additionally profiled by comparing contig sequences against the Virulence Factor Database (VFDB; Liu et al., 2019) using default parameters and cut-off threshold of 90% identity and coverage. The ABRicate tool v.1.0.0 (Seemann, 2019) was used against the built-in ResFinder, CARD, and VFDB databases to corroborate the identity of all ARG/virulence factors identified.

#### 2.3.5. Functional prediction

For functional prediction of the metabolic pathways influenced by sub-MIC antibiotics, the HUMAnN v.3.1.1 pipeline (Beghini et al., 2021)^2^ was used. Subsampled, trimmed paired reads were concatenated into a single file per sample and mapped against the ChocoPhlAn (microbial database) and the full UniRef (version: uniref90_201901b) reference databases for gene family identification. Default parameters were used with the “—taxonomic-profile” option, since the previously generated taxonomic profile output from MetaPhlAn was used as a custom taxonomic profile for the analysis. HUMAnN’s default curated metabolic pathway database, MetaCyc (Caspi et al., 2018) was used for pathway annotation. The abundance of each pathway (path_abundance.tsv file) of all samples was merged into a single table using the “humann_join_tables” option. The list of each identified pathway is represented in reads per kilobase (RPK) units with the abundance of each pathway categorized into bacterial organisms (in HUMAnN referred to as “stratifications”). After filtering out unidentified functional pathways, a pathway abundance table was used for subsequent multivariate and statistical analyses. To generate coverage information of calculated functional genes and ARG sequences, trimmed paired reads (.fastq) were mapped against the contigs of each metagenome assembly using Bowtie2 v. 2.4.4 (Langmead and Salzberg, 2012) with -bowtie2-build and default settings. Then, Samtools v.1.15.1 (Li et al., 2009) was used to visualize a file report of sequence coverage across samples.

### 2.4. Statistical analysis

All statistical analyses were performed in R version 4.2.0 (R Core Team, 2020). One-way ANOVA was used when normality assumptions were met (tested using Tukey HSD, for instance, for microscopic analyses). For non-parametric data, Kruskal-Wallis was applied to untransformed data to test for significant differences (*p* < 0.05) in biofilm composition, biomass abundance percentage, and diversity indices. *p*-values were adjusted using the Benjamini-Hochberg method to reduce false positive results (*p*adj < 0.05; Benjamini and Hochberg, 1995). Diversity indices were calculated based on the untransformed abundance data of bacterial biofilm communities at genus and species levels. Species richness (Chao1), Shannon and Simpson indices were estimated within samples to describe α-diversity (Supplemental Figures S3, S4).

Comparisons between control and sub-MIC treatment-exposed communities were assessed through permutational analyses of variance based on Bray–Curtis dissimilarity indices (PERMANOVA, 999 permutations) calculated from Hellinger-transformed abundance count tables of the microbiome or resistome using *vegdist* function with vegan package v. 2.6.2 (Oksanen et al., 2020). Pairwise comparisons between control and treatments were performed to determine significance (*p* < 0.05). Bray-Curtis distance matrices were used to calculate β-diversity between samples. β-diversity was visualized with non-metric multidimensional scaling plots (nMDS) to ordinate microbiome and resistome data. Significant dissimilarity of ordination between groups was assessed using the Analysis of Similarities statistic (ANOSIM). To screen for significant differences (log^2^-fold changes) in gene and functional pathway abundance, the DESeq2 v.1.35 (Love et al., 2014) package was used, where the *contrast* function was employed to extract values per sub-MIC antibiotic condition.

The Bray-Curtis distance matrices resulting from the nMDS ordination were visualized for correlation patterns between the microbiome and resistome through the *procrustes* function in the vegan package. The Spearman’s rank correlation via Mantel test was used to compare the similarity of the microbiome and resistome databases, and to assess the relationship between the microbiome and the resistome in the presence of sub-MICs antibiotics, where correlations considered to be significant (*p* < 0.05) and with a dissimilarity coefficient > 0.75 were visualized in network analysis using Hmisc v.4.7-2 and igraph package v.1.3.5.

## 3. Results

### 3.1. Structural biofilm composition

**Figure 1:**
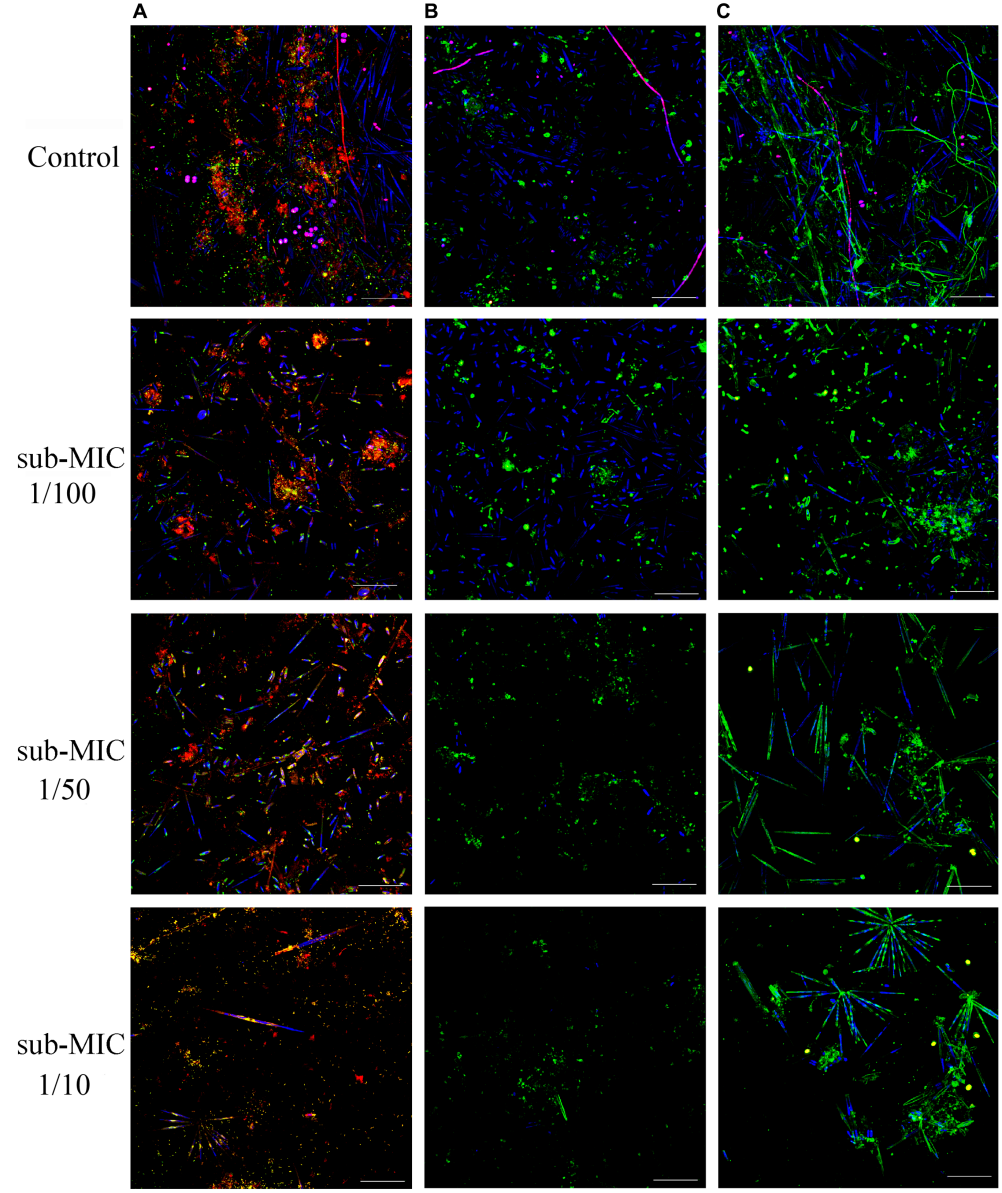
CLSM stacked image results of biofilms grown without or in the presence of sub-MICs antibiotic exposure after 8 weeks of development. Samples were stained by **(A)** *Triticum vulgaris*—TRITC lectin and SYTO9 showing EPS (red), bacteria (yellow), algae (blue) and cyanobacteria (magenta), **(B)** *Arachis hypogaea*—FITC and **(C)** *Canavalia ensiformis*—FITC lectins showing EPS (green), algae (blue), and cyanobacteria (magenta). Scale bar indicates 50 μm.

Riverine biofilm structure was altered following sub-MICs antibiotic exposure. CLSM stacked images from microscopic analysis showed differences in biofilm architecture across treatments (Figure 1). Overall, sub-MICs antibiotic exposure decreased the complexity of biofilm communities, with the sub-MIC 1/10 treatment having the most pronounced effects on overall biofilm architecture, including biofilm thickness, and biomass percentage of bacteria, autotroph, and EPS composition. While pennate diatoms were observed in all samples, cyanobacteria (magenta fluorescence) were only recorded in control biofilms (Figure 1).

**Figure 2:**
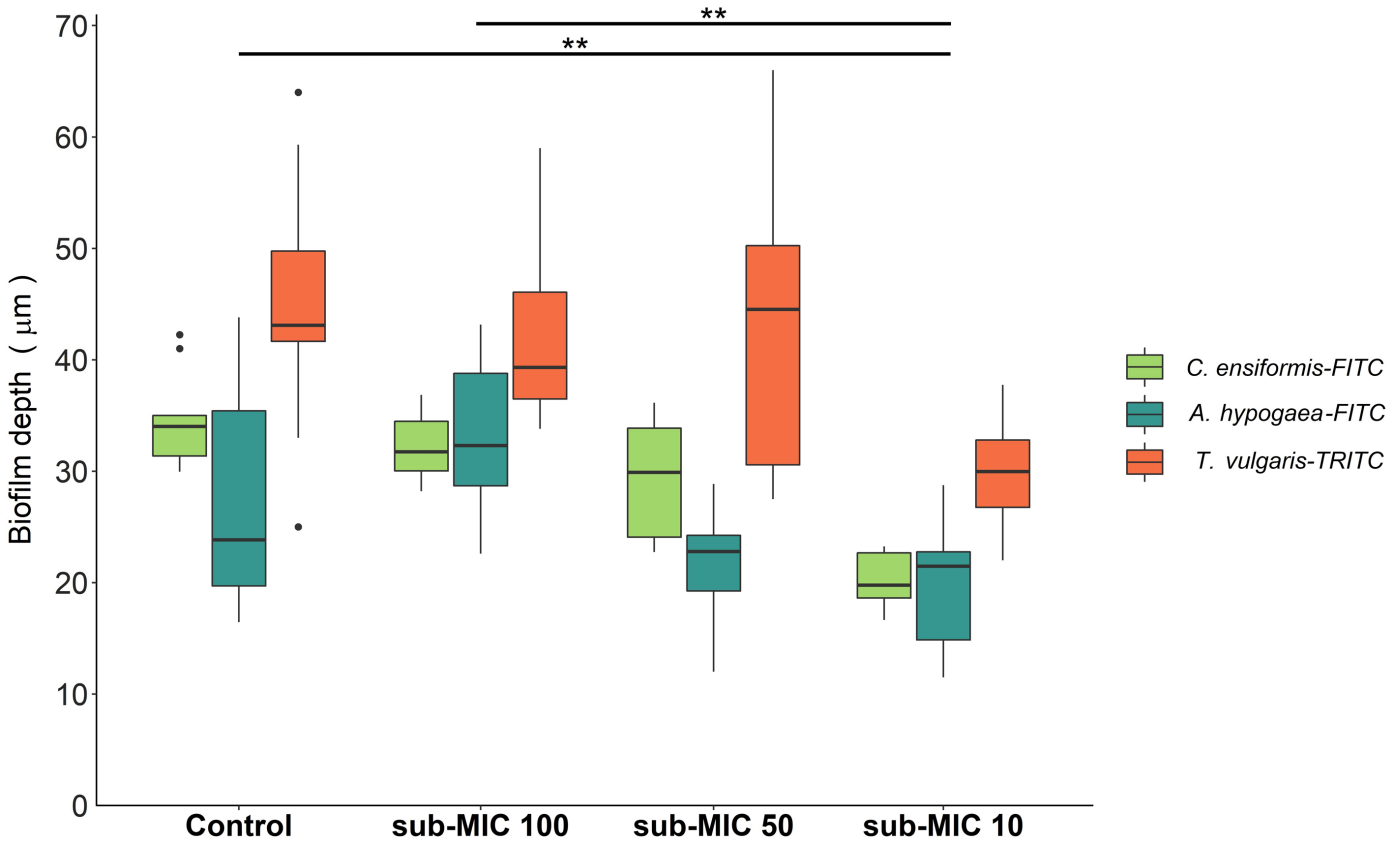
Biofilm thickness of microbial communities under sub-MICs antibiotic exposure was based on fluorescence of lectin-binding specificity to EPS glycoconjugate residues. Mean values are displayed with biological (*n* = 3) and technical (*n* = 5) replicates (**p < 0.05). Edges represent quartile values.

Biofilm thickness was calculated based on the fluorescence emitted by the lectin probes: *C. ensiformis*-FITC, *A. hypogaea*-FITC and *T. vulgaris*-TRITC (Figure 2) which target the EPS matrix associated with diverse microbial community members and are commonly used as indicators of changes within biofilm communities (Lawrence et al., 2007b, 2009). Decreased biofilm thickness was consistently observed with increasing strength of antibiotic treatment. The sub-MIC 1/10 treatment resulted in significantly decreased biofilm thickness relative to controls and the lowest (sub-MIC 1/100) treatment (*F* = 12.3, *p* < 0.05; Figure 2). sub-MIC 1/50 and sub-MIC 1/100 treatments resulted in thinner biofilms compared to control samples, though not significantly so.

**Figure 3:**
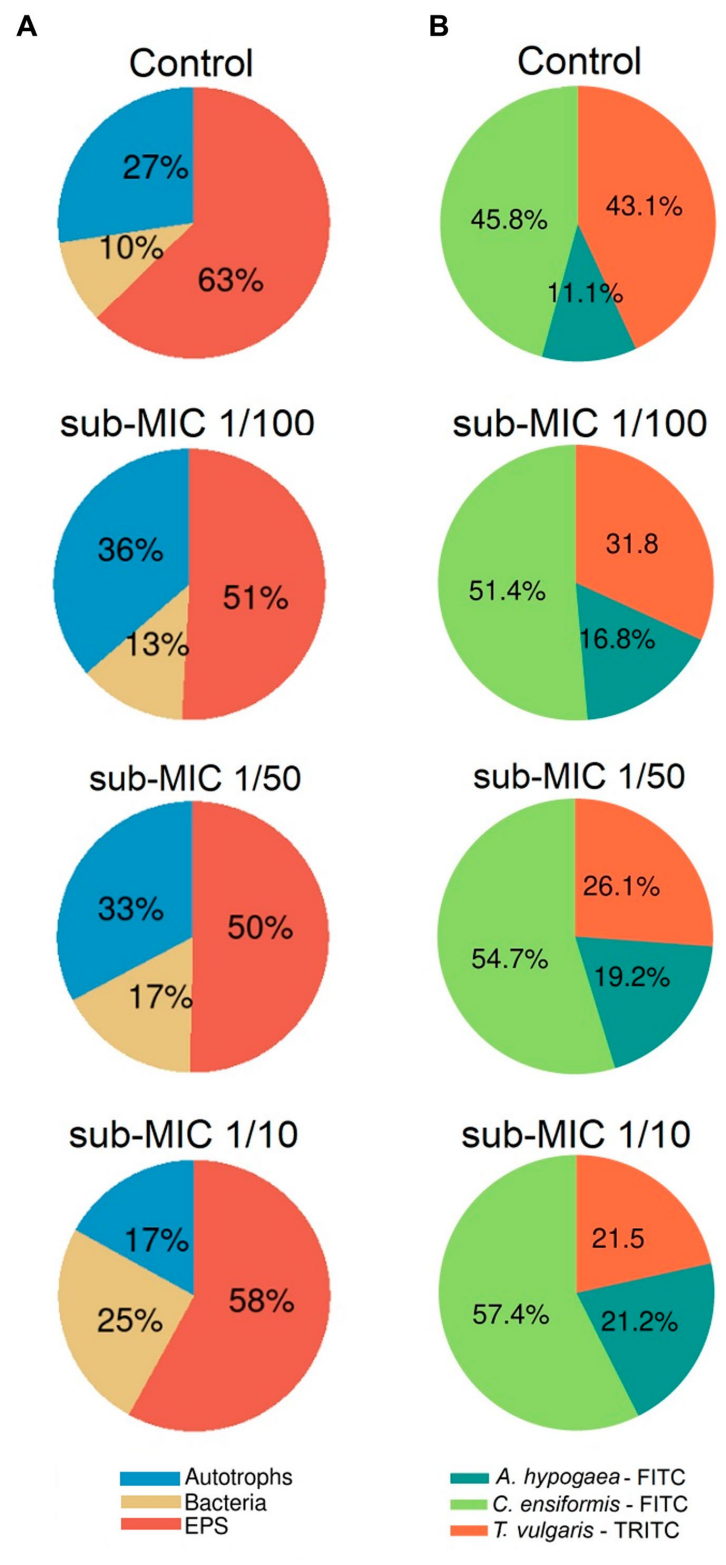
Proportional abundance of biofilm architecture elements exposed to sub-MICs antibiotics. **(A)** Relative biomass abundance of bacteria, EPS and autotroph composition, and **(B)** Relative lectin-binding specificity of three probes used to characterize the glycoconjugate composition of the EPS matrix. Proportional abundance is the coverage percentage recorded of the fluorescent signal of each biofilm structure element from Z-stack images. Mean values are displayed with biological (*n* = 3) and technical (*n* = 5) replicates.

The EPS characterized by the glycoconjugate distribution showed residues of mannose, glucose and *N-*acetylglucosamine through all biofilm samples, which are carbohydrate residues typically found in freshwater biofilms (Dynes et al., 2009; Flemming et al., 2022). The EPS biomass percentage, as measured by the fluorescent signal from the three lectin probes, significantly shifted (*F* = 8.8, *p* < 0.05) between samples (Figure 3B). *Canavalia ensiformis*-FITC and *A. hypogaea*-FITC signal percentage increased in the presence of sub-MICs antibiotics, whereas *T. vulgaris*-TRITC lectin binding to *N*-acetylglucosamine residues and oligomers decreased. Exopolymer composition throughout all samples was dominated by *C. ensiformis*-FITC binding to mannose and glucose residues, with an increase in fluorescence (binding specificity) after sub-MICs antibiotic exposure, and most notably observed in the sub-MIC 1/10 treatment.

Proportional biomass abundance changes in the bacterial, autotroph and glycoconjugate composition (EPS matrix) were calculated based on analysis of the fluorescence of the three-channel images (Figure 3A). The autotroph proportional biomass amount (from signals of both prokaryotic and eukaryotic autotroph species) was significantly different between treatments (*F* = 4.9, *p* < 0.05) and was highest in the sub-MIC 1/100 treatment (36%), followed by the sub-MIC 1/50 treatment (33%), control samples (27%) and the sub-MIC 1/10 treatment (17%). Overall, EPS biomass significantly decreased (*F* = 12.2, *p* < 0.05) after sub-MICs antibiotic exposure relative to control values (63%), with the lowest biomass EPS composition detected in the sub-MIC 1/50 treatment (Figure 3A). Interestingly, CLSM image analysis did not show significant differences in the amount of bacterial biomass, although highest proportion was observed in the sub-MIC 1/10 treatment (25%), followed by the sub-MIC 1/50 treatment (17%), the sub-MIC 1/100 treatment (13%), and lastly the control samples (10%).

The cumulative number of protozoa was also tracked (Supplementary Figure S1), revealing that ciliates dominated control biofilms and overall protozoa counts significantly decreased (*x^2^* = 7.8, *p* < 0.05) in the presence of sub-MICs antibiotics. However, no correlations, or more specific protozoan predation or grazing pattern interactions between biofilms developed under antibiotic concentrations (sub-MIC 1/10, 1/50 and 1/100), were detected. Despite the heterogeneous biofilm communities and thickness range, overall visualization of the EPS matrix confirmed that control biofilms contained thicker EPS than sub-MICs treated communities, yet conversely, bacterial biomass increased with sub-MICs antibiotic treatments. These results, in combination with decreased protozoan and cyanobacteria, indicate a selective effect of sub-MICs antibiotics on biofilm communities.

### 3.2. Microbiome of riverine biofilm communities

**Figure 4:**
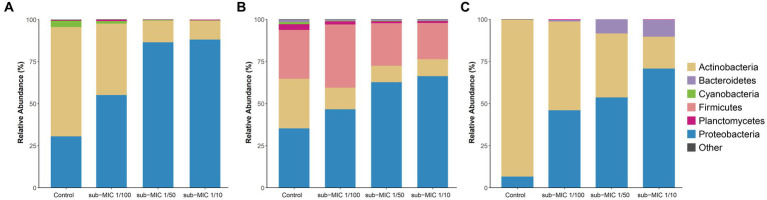
Comparison of taxonomic classification tools. Relative bacterial sequence abundances for **(A)** CosmosID, **(B)** Kraken, and **(C)** MetaPhlAn tools at the phylum level (*n* = 3).

The bacterial community composition was analyzed by using three commonly utilized bioinformatic tools for metagenome taxonomic profiling: CosmosID, Kraken and MetaPhlAn. Across the three approaches, the taxonomic profile of the biofilm shifted in response to sub-MICs antibiotic treatment, with Actinobacteria and Proteobacteria being the predominant phyla groups, constituting more than 70% of overall community across all samples (Figure 4). Interestingly, the proportion of Proteobacteria was lowest in the control samples and increased along with sub-MIC antibiotics concentration. In contrast, Actinobacteria species were consistently reduced in the presence of sub-MIC antibiotics, as were abundances of Bacteroidetes, Cyanobacteria and Planctomycetes. The phylum Firmicutes was only detected via Kraken, with an average relative abundance of 30% and little variation across samples.

**Figure 5:**
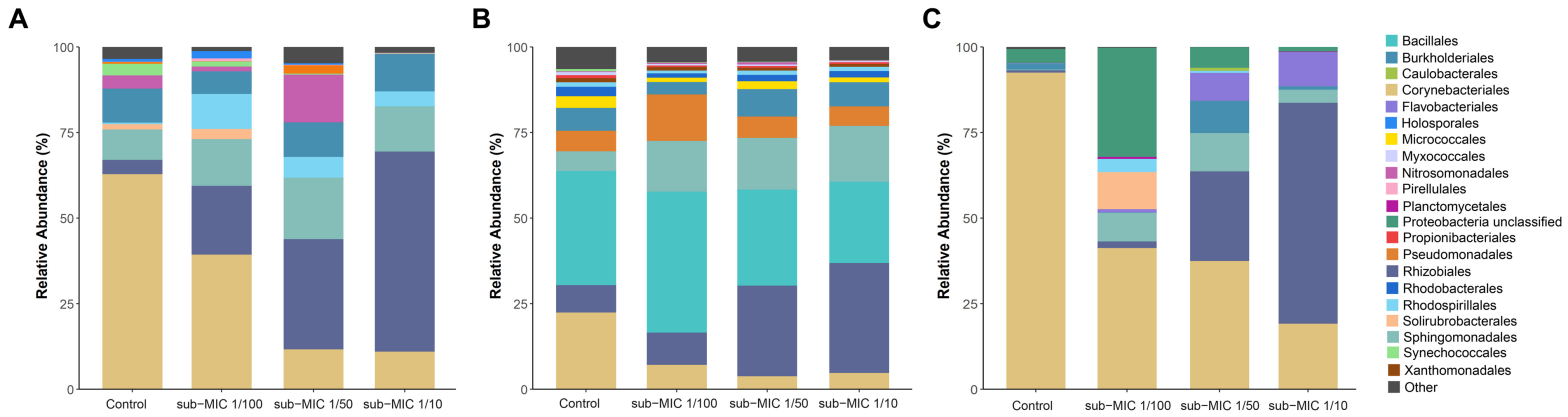
Comparison of taxonomic classification tools. Relative bacterial sequence abundances for **(A)** CosmosID, **(B)** Kraken, and **(C)** MetaPhlAn tools at the order level (>1%; *n* = 3).

While there were notable differences in the taxonomic profiles produced by the three different identification databases, most identified taxa were common across tools. A total of 11 different classes (Supplementary Figure S2) and 20 order groups (Figure 5) were identified with >1% relative abundance across samples. At the class level, bacterial abundance changes were mostly associated with Actinobacteria and Alphaproteobacteria, the former decreasing at the highest treatment concentration (sub-MIC 1/10). All tools showed similar abundance trends at the order level for Burkholderiales, Corynebacteriales, Sphingomondales, and Rhizobiales. The Bacilli class and Bacillales order were only observed in Kraken, constituting 25%–30% of relative abundance.

**Figure 6:**
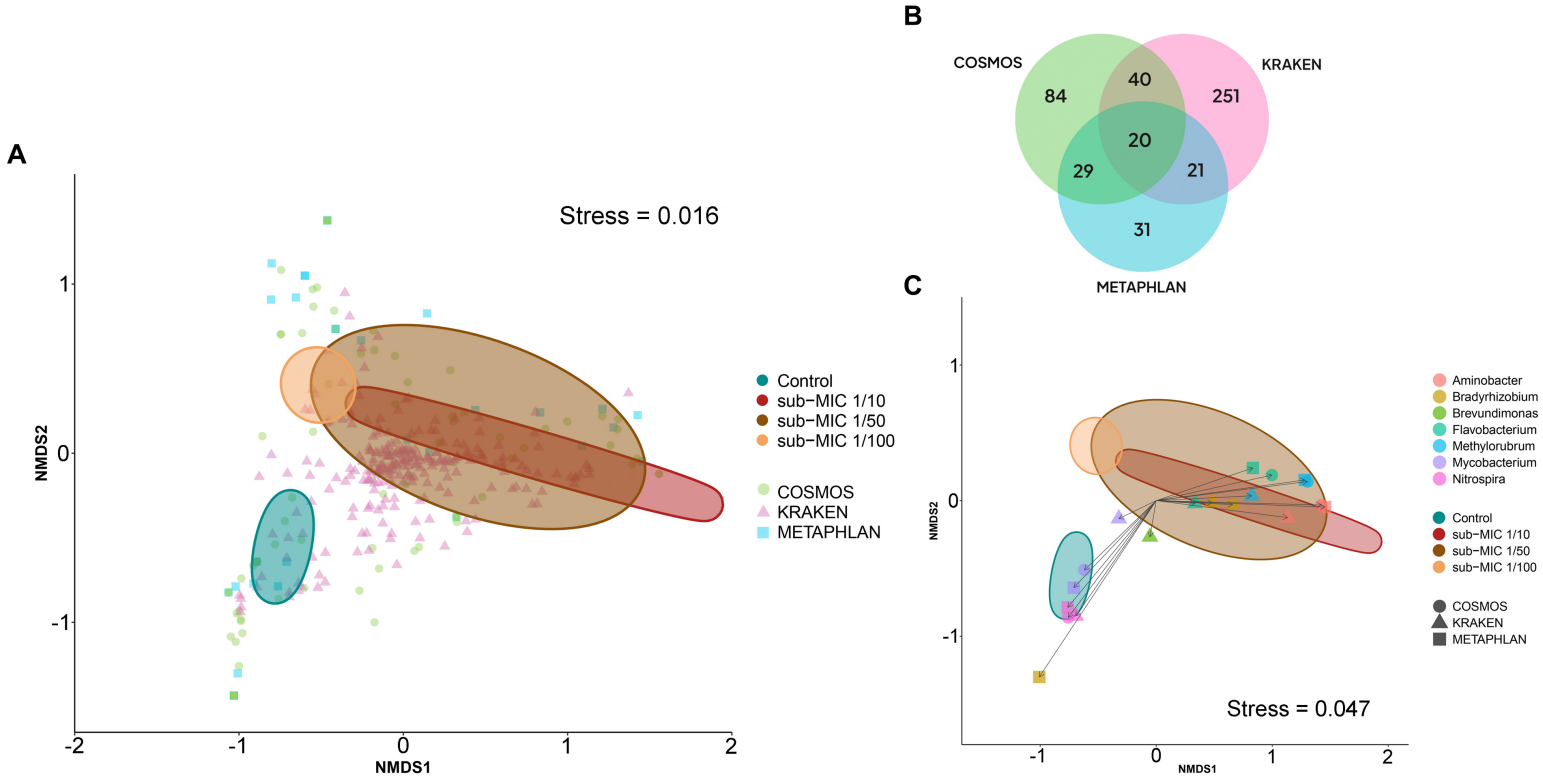
Bacterial composition at the genus level according to three taxonomic databases of riverine biofilm communities cultivated under sub-MICs antibiotic exposure. **(A)** nMDS two-dimensional plot of Bray-Curtis similarities showing β-diversity identified in the three taxonomic databases: CosmosID (84 = genera), Kraken (251 = genera) and MetaPhlAn (31 = genera). **(B)** Venn diagram displaying identified total genera groups across taxonomic databases. Overlap indicates the number of shared taxa among databases. **(C)** nMDS plot of Bray-Curtis similarities of significant genera (*p* < 0.001) detected at each taxonomic database. Ellipses shape is defined by covariance of each group, and ellipses centroid represents the group mean.

At the level of genus and species, profile differences were observed across the identification tools. CosmosID analysis detected 84 different genera, and 100 bacterial species; MetaPhlAn identified 31 genera and 39 species; and Kraken detected 251 genera and 2,572 species (Figure 6B). Comparison of these tools showed that MetaPhlAn was less sensitive for identifying bacteria at species level (most groups were categorized as unclassified), whereas Kraken showed high sensitivity to species strains. α-diversity showed markedly different trends according to the taxonomic pipeline used. Genus-level richness was determined to be higher in control biofilms relative to sub-MIC samples, except for MetaPhlAn results; however, significant differences were only observed between the 1/100 antibiotic treatment and control samples (Kruskal-Wallis *x^2^* = 8.4, *p* < 0.05, Kraken, Supplementary Figure S4). Species level-richness was consistently lowest in the sub-MIC 1/100 treatment, although the analysis did not demonstrate significant differences across samples (Supplementary Figure S5).

Despite the observed differences, Shannon and Simpson indices calculated from all databases demonstrated higher genera diversity of biofilms at the sub-MIC 1/50 treatment compared to the sub-MIC 1/10 condition. Species-level diversity yielded consistently similar diversity distributions, but just for the Shannon index. Overall, calculated genus and species-level α-diversities from CosmosID and MetaPhlAn were more similar to each other than to the Kraken results. For example, diversity of control biofilms relative to sub-MIC treatments were less diverse in MetaPhlAn and CosmosID analyses, yet control samples in the Kraken analysis, the most species-sensitive package, were the most taxonomically diverse, with significant differences when compared to sub-MIC 1/10 treated communities (*x^2^* = 7.8, *p* < 0.05; Supplementary Figure S4).

The Bray-Curtis distance matrices of identified bacteria at genus level were used to generate nMDS plots to assess the dissimilarities between biofilm microbial communities developed under different sub-MIC antibiotic exposures (Figure 6A). The three taxonomic tools displayed significant differences across sub-MIC antibiotic treated samples (CosmosID: R^2^ = 0.577, *p* = 0.017; Kraken: R^2^ = 0.609, *p* = 0.040; MetaPhlAn: R^2^ = 0.537, *p* = 0.018). Of the total 296 unique genus groups identified across taxonomic analyses, only 20 were shared across the three databases (Figure 6B). Interestingly, seven genera were shown to be significant across all three taxonomic tools (PERMANOVA*, F =* 3.6*, p* < 0.001, Bray-Curtis distance), suggesting their abundances drove the ordination pattern observed among biofilm communities (Figure 6C) *Mycobacterium* and *Nitrospira* genera were associated with control samples, whereas *Flavobacterium, Methylorubrum* and *Aminobacter* genera were associated with biofilm communities cultivated under sub-MIC 1/10 and sub-MIC 1/50 conditions.

Correlation analysis between the taxonomic databases indicates that identified genera from the CosmosID and MetaPhlAn databases shared the most similarities in taxonomic composition when examining genus-level abundance (Spearman’s rho = 0.895; Supplementary Figure S7A). Comparison of the genera abundance from the three databases was significant (*p* < 0.05), but Mantel results from CosmosID vs. Kraken (Spearman’s rho = 0.711) and MetaPhlAn vs. Kraken (Spearman’s rho = 0.644) were only moderately correlated.

### 3.3. Biofilm community resistome and virulome trends

**Figure 7:**
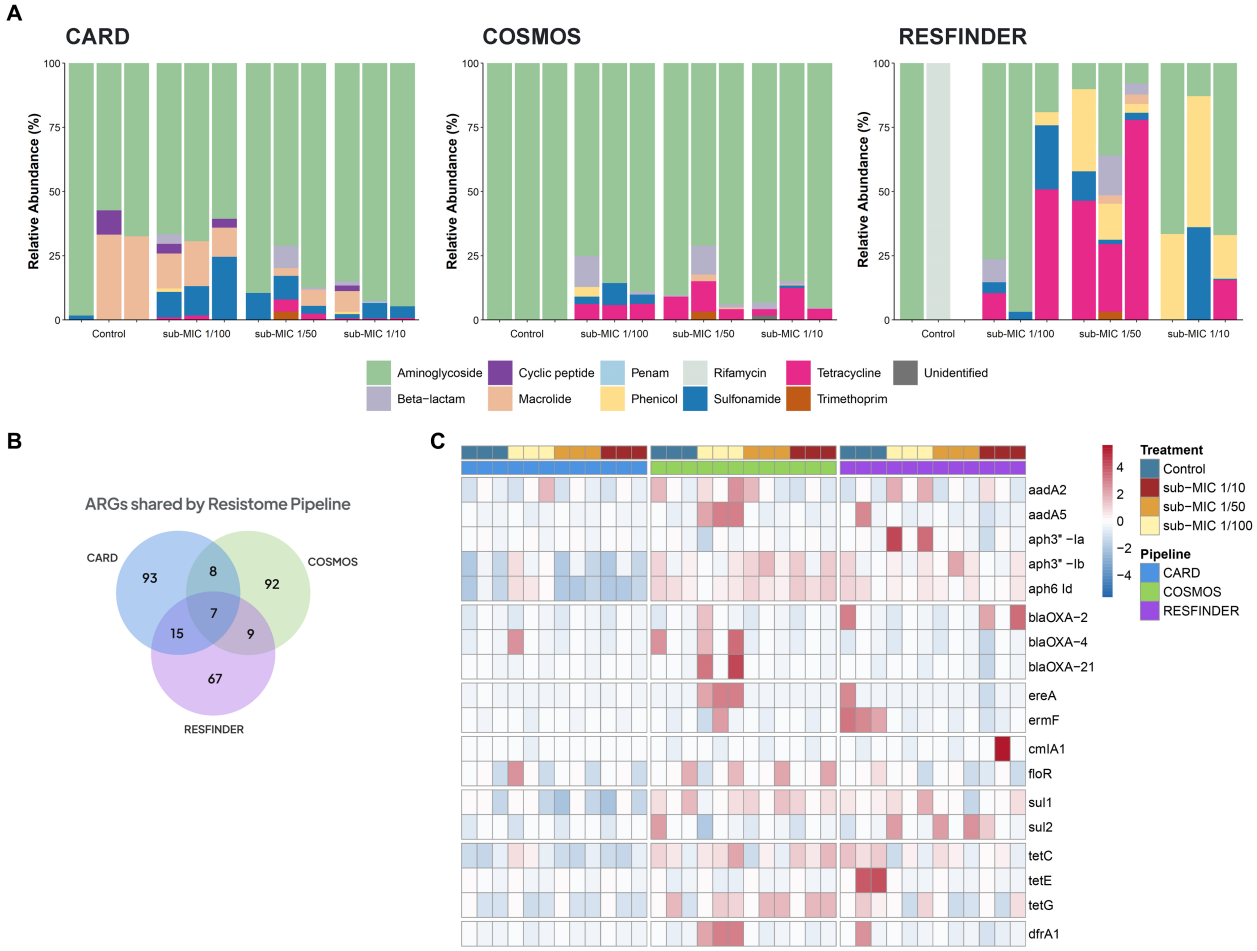
ARGs composition according to the resistome database in riverine biofilm communities under sub-MICs antibiotic exposure. **(A)** Relative abundance classified by drug class resistance identified in each database. **(B)** Venn diagram displaying total identified number of ARGs. Overlap indicates the number of shared ARGs across databases. **(C)** ARGs with significant fold-changes (*p* < 0.05) between treatments. The normalized abundance heatmap is scaled to each gene (Z-score) after rlog-transforming counts.

Three different antibiotic resistance databases were compared to determine the resistome of riverine biofilm communities under sub-MIC antibiotic exposure. Across all biofilm community samples, a total of 92 ARGs were detected via CosmosID, 93 ARGs in CARD, and 67 ARGs in the ResFinder database (Figure 7B). Normalized abundance of ARGs determined from the three database analyses demonstrated a marked increase in the copy numbers of ARG in samples under sub-MIC exposure relative to control samples (Supplementary Figure S3).

Resistome composition from the CosmosID pipeline consisted of 33 different types of ARGs corresponding to seven drug resistance classes: aminoglycosides, β-lactams, macrolides, phenicols, sulfonamides, tetracyclines and trimethoprim. ARGs from the aminoglycoside drug class represented the majority of the resistome, followed by tetracycline and β-lactam genes (Figure 7C). Results from the CARD database detected 24 different types of ARGs which were grouped into nine drug resistance classes: aminoglycosides, β-lactams, cyclic peptides, macrolides, penams, phenicols, sulfonamides, tetracyclines and trimethoprim. CosmosID, relative abundances showed ARGs from aminoglycoside drug class to be the predominant group across control and sub-MICs exposed samples; however, macrolide and sulphonamide ARGs were the next most-abundant genes (Figure 7A). The ResFinder database identified 25 different types of ARGs across samples (Figure 7A) within eight different drug classes: aminoglycosides, β-lactams, macrolides, phenicols, rifamycins, sulfonamides, tetracyclines, and trimethoprim. However, no ARGs in the ResFinder database with an identity above the 90% identity cut-off threshold were detected in any of the control biological replicates.

Several ARGs were detected in at least two of the resistome databases (Figure 7B), with all three pipelines predicting the same seven ARGs under sub-MIC conditions (Supplementary Table S3). Aminoglycoside resistance was present at greater than 70% relative abundance through all samples according to CosmosID and CARD databases and were the predominant ARGs group in the ResFinder analysis. The *aph6-Id* aminoglycoside gene was prevalent throughout all samples and across all three pipelines. When examining ARGs that were more abundant in sub-MIC exposures relative to control samples (log^2^FoldChange, *p*adj *<* 0.05), all three gene resistance databases predicted 18 common ARGs consisting of the classes aminoglycosides, β-lactams, macrolides, sulphonamides, tetracyclines and trimethoprim (Figure 7C). Most notably, gene copies of *aadA*5*, drfA1, ereA,* and *ermF* were highest at the sub-MIC 1/50 treatment, whereas *sul*2 was highest at the sub-MIC 1/100 treatment.

We further measured the richness of the resistome in all samples classified by gene, drug class, and mechanism of resistance (Supplementary Figure S6). Across the three profiled pipelines, microbial communities developed under sub-MICs antibiotic exposure displayed higher absolute numbers of ARGs with gene richness significantly greater than control samples (*H*=, *p <* 0.05). Annotations performed by the CosmosID pipeline showed that the seven drug classes and four mechanisms of antibiotic resistance were significantly increased in sub-MIC treatments relative to controls. The CARD pipeline revealed significant differences between sub-MIC 1/10 and 1/100 treatments relative to control samples only at the mechanism level, but not at the gene or drug class level. Results from the ResFinder pipeline showed significant differences between resistome richness of the sub-MIC 1/50 treatment relative to control samples at the level of individual ARG, drug class, and mechanisms of resistance (Supplementary Figure S6). ARGs from the CARD database displayed a significant correlation with the ResFinder database (Spearman’s rho = 0.556, *p* = 0.004). However, the relationships between the other databases showed non-significant and weak associations with identified ARGs (Supplementary Figure S7B).

**Figure 8:**
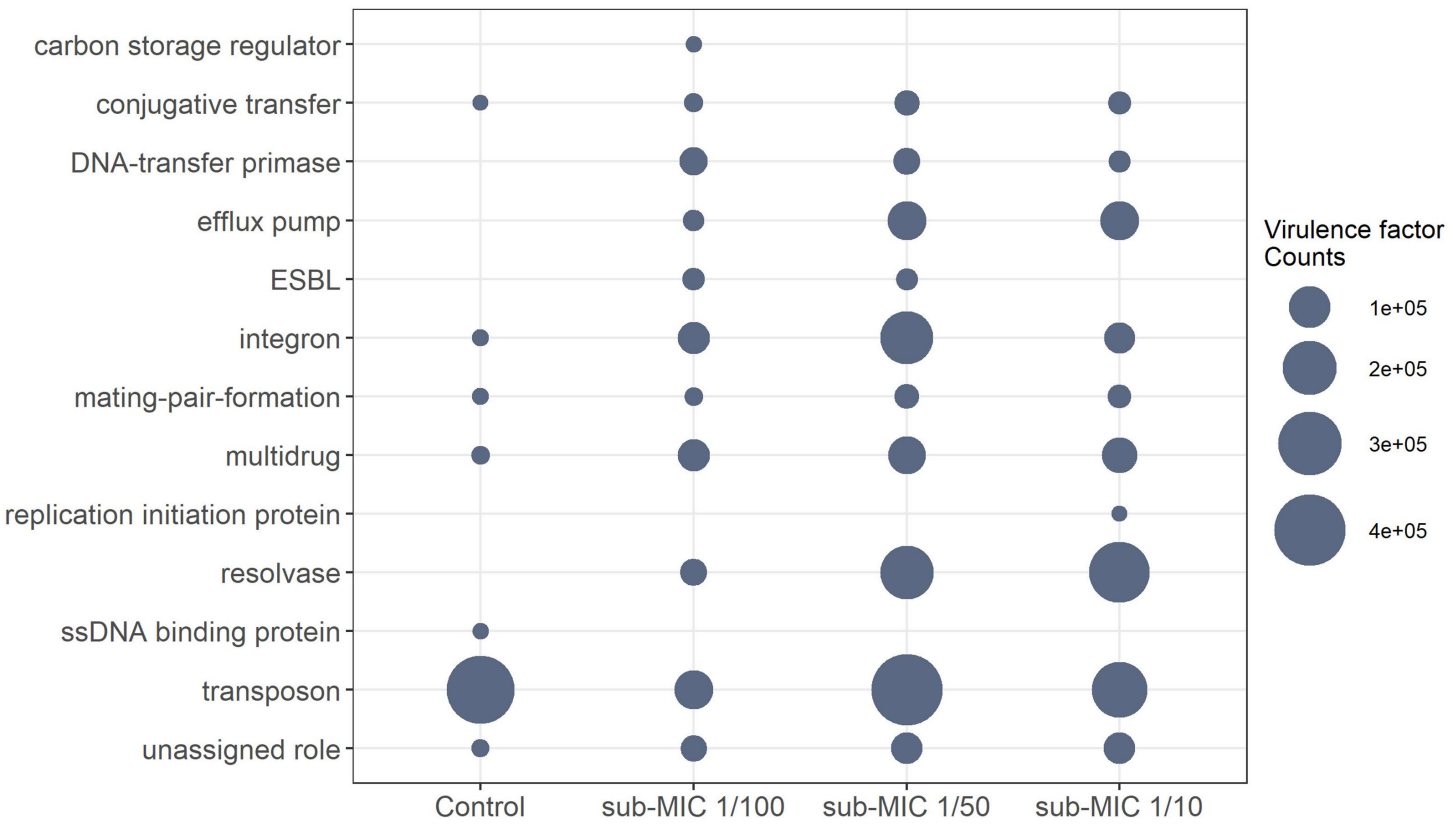
Differential abundance of normalized virulence factor comparisons with significant differences between sub-MICs antibiotic treatments (*p* < 0.05) using Benjamini-Hochberg correction for multiple comparisons.

The virulome of riverine biofilm communities was also analyzed, since it is known that pathogenic or non-pathogenic bacteria can acquire “accidental virulence” as result of the advantageous conditions within biofilm niches (Andersson et al., 2018). Virulence genes associated with conjugative transfer, efflux pumps, DNA-transfer primases, integrons, mating-pair-formation, resolvases, transposons, and multidrug factors, were detected across the three sub-MIC antibiotic conditions (Figure 8; Supplementary Figure S8). Virulome composition was shown to be significantly different across sub-MIC conditions (*x^2^* = 8.9, *p* < 0.05). A total of 89 genes associated with virulence were identified across all samples, among which transposon genes (i.e., *TpnA*) and multidrug resistance genes (i.e., *sul1* and *qacEdelta1*) were not only observed in control samples but were also the most abundant type of virulence factors according to the VFDB database (Figure 8). Samples from sub-MIC 1/10 and 1/50 treatments showed very similar virulome compositions. However, extended-spectrum β-lactamase (ESBL) genes, such as *blaOXA*-1 and *blaOXA*-2, were associated with sub-MIC 1/50 and 1/100 antibiotic samples. Moreover, *ssb*, a gene coding for single-stranded DNA-binding protein, was only detected in one control replicate.

### 3.4. Biofilm community functional capacity

**Figure 9:**
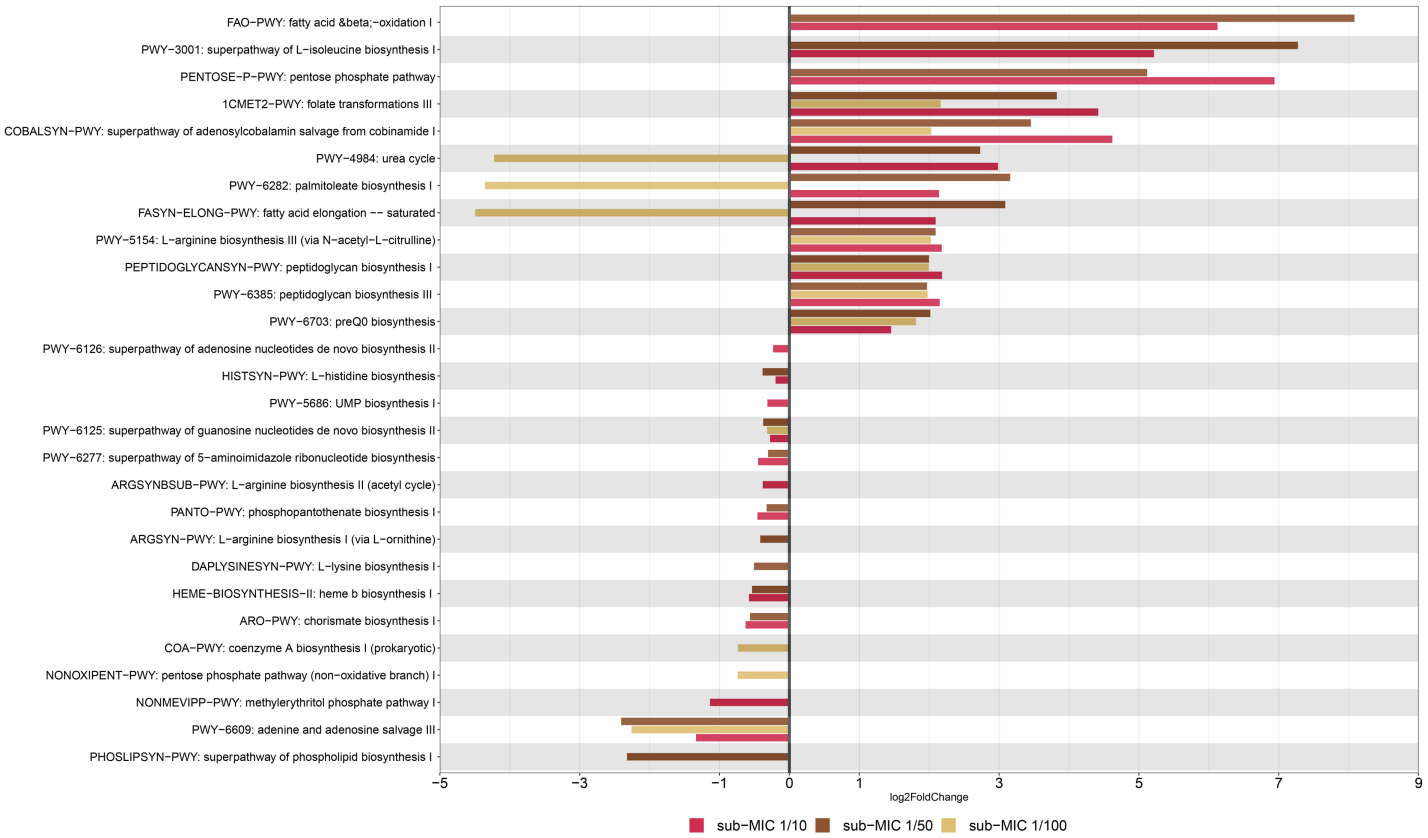
Functional profiling of riverine biofilm communities under sub-MICs antibiotic exposure. Y-axis represents metabolic pathways annotated by MetCyc from HUMAnN analysis; and X-axis represents significant differential abundance sub-MICs antibiotic condition relative to control samples using DESeq2 (*p* < 0.05).

The prediction of metabolic pathways in riverine biofilms revealed a total of 106 functional pathways present throughout samples. “Unidentified” pathways and those that confer functionality to non-bacterial organisms (such as plants or mammals) were manually removed prior to statistical analysis and visualization. Differential gene abundance changes of 99 pathways were analyzed via DESeq2, yielding 28 significant functional pathways related to sub-MIC antibiotic treatments relative to control samples (Figure 9). In general, detected bacterial metabolic pathways were related to biosynthesis and nucleotide salvage. Following the nomenclature by the MetaCyc database, these biosynthesis pathways included the super-classes for nucleoside and nucleotide biosynthesis (PWY-5686, PWY-6126, PWY-6125, PWY-6277, PWY-6609), phosphate-related pathways (NONOXIPENT-PWY, PENTOSE-P-PWY), cell wall biosynthesis (PWY-6385, PEPTIDOGLYCANSYN-PWY), fatty acid and lipid biosynthesis (PHOSLIPSYN-PWY, FASYN-ELONG-PWY, PWY-6282), secondary metabolite biosynthesis (NONMEVIPP-PWY, PWY-6703), Cofactor, carrier and vitamin biosynthesis (PANTO-PWY, COBALSYN-PWY, 1CMET2-PWY, COA-PWY, HEME-II), and aromatic compound biosynthesis (ARO-PWY).

Among genes associated to superclasses for amino-acid biosynthesis, L-isoleucine (PWY-3001) and L-arginine (PWY-5154) were more abundant under sub-MIC antibiotic exposure compared to control samples; yet L-histidine (HISTSYN-PWY), L-arginine (ARGSYN-PWY, ARGSYNBSUB-PWY) and L-lysine (DAPLYSINESYN-PWY) displayed modest decreases in differential gene abundance (log^2^ Fold-change < −1., *p* < 0.05) in sub-MIC antibiotic conditions, except for sub-MIC 1/100 (Figure 9). Interestingly, genes related to arginine biosynthesis via acetyl-L-citrulline showed two log^2^ Fold-change higher abundance in the three sub-MIC antibiotic treatments; however, genes related to arginine biosynthesis via the acetyl cycle and the L-ornithine pathway were less abundant (−0.5 log^2^ Fold-change) in the sub-MIC 1/10 and sub-MIC 1/50 treatments, respectively. Overall, genes of functional pathways involved in biosynthesis showed negative differential abundances under exposure to sub-MIC treatments, with abundance fold-change varying across treatment. Several genes involved in functional pathways showed significant relative abundance differences (*p* < 0.05) between sub-MIC 1/10 (23 pathways) and sub-MIC 1/50 (22 pathways) treatments relative to controls, although the sub-MIC 1/100 treated samples showed fewer significantly differentiated metabolic pathways (13 pathways).

Nucleoside and nucleotide biosynthesis pathways demonstrated lower relative gene abundances (log^2^ Fold-change −2.40 to −0.23., *p* < 0.05) at all sub-MIC treatments in comparison to control samples. In contrast, two cell wall biosynthesis pathways showed higher abundances across the three sub-MIC antibiotic treatments (log^2^ Fold change1.9–2.2, *p* < 0.05) relative to controls. Notable degradation pathways were assigned to the general fatty-acid pathway (FAO-PWY), which was present in sub-MIC 1/10 and 1/50 treatments, and the urea cycle (PWY-4984). The urea cycle and fatty acid (PWY-6282, FASYN-ELONG-PWY) pathways had positive abundance shifts for sub-MIC 1/10 and sub-MIC 1/50 treatments but negative shifts for the sub-MIC 1/100 treatment relative to control biofilms. Notably, urea degradation is commonly associated to *Nitrospira* spp.; however, this genus was most strongly associated with the control biofilm condition, suggesting nitrogen and urea cycling are not clearly impacted or linked to sub-MIC treatments. Functional pathways with the most pronounced abundance shift between treatments were illustrated according to the contributing bacterial genera (Supplementary Figure S9). The bacterial species associated with detected functional pathways included the genera *Afipia, Aminobacter, Blastomonas, Bosea, Bradyrhizobium, Caulobacter, Flavobacterium, Sphingomonas, Methylibium, Methylorubrum, Mycobacterium, Mycobacteroides*, and *Variovorax*.

### 3.5. Microbiome and resistome relationship

**Figure 10:**
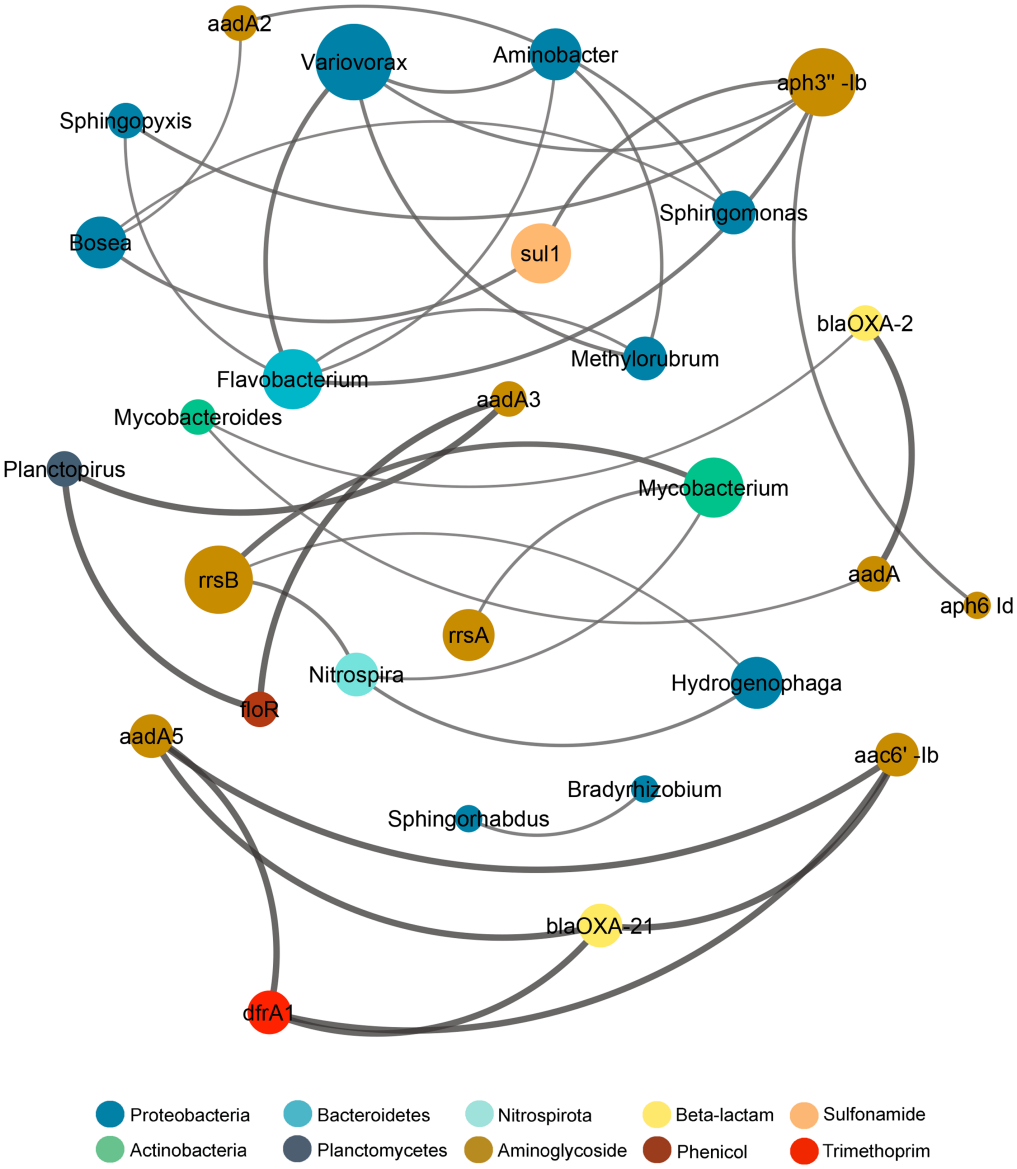
Network analysis showing correlation patterns between the microbiome and resistome after sub-MICs antibiotics exposure. Edges (lines) connecting nodes mean a strong correlation (Spearman’s rho > 0.75, *p* < 0.05), negative indicated in red and positive in gray. The size of each node is proportional to the number of connections (degree; *n* = 12).

The co-occurrence relationship between the microbiome and the resistome composition was assayed through the Procrustes analysis by using the Mantel test and Procrustes test for statistical validation (Supplementary Figure S10). Both tests showed significant correlation between the microbiome and resistome dissimilarity matrices (Mantel r = 0.652, *p* = 0.001; Procrustes M^2^ = 0.257, r = 0.861, *p =* 0.001), thus demonstrating that the bacterial and resistome composition were closely associated. Since the Procrustes analysis only illustrates overall correlations, a network analysis was performed to further examine individual co-occurrence patterns between the microbiome and resistome in biofilm communities after sub-MIC antibiotic exposure (Figure 10). The network analysis was based on strong and significant correlations (Spearman’s rho > 0.75, *p <* 0.05) between a correlation matrix constructed with 23 ARGs and 20 bacterial taxa (genus level).

Additionally, co-occurrence analysis indicating resistome and microbiome patterns for each of the sub-MIC treatments (1/10, 1/50, and 1/100) was performed (Supplementary Figure S11). For details about the networks’ topological properties, such as the number of edges between nodes of each network, see Supplementary Tables S5, S6. Based on the connections between nodes, the most densely connected nodes were regarded as indicators of co-occurrence of ARGs. Microbiome composition results showed that *Aminobacter*, *Bosea*, *Flavobacterium*, *Sphingomonas* and *Variovorax* displayed the highest co-occurrence (4 nodes) to microbial and resistome nodes. Aminoglycoside ARGs (*aph3”-Ib, aadA2*, *rrsB*) demonstrated co-occurrence to Proteobacteria genera, but also to Actinobacteria (*aadA*, *rrsA*, *rrsB*), Bacteroidetes (*aph3”-Ib*) and Nitrospirota (*rrsB*; Figure 10). Indeed, Proteobacteria genera, such as *Bradyrhizobium, Methylorubrum, Novosphingobium, Sphingobium*, and *Variovorax,* correlated with *aph3”-Ib*, *aph6-Id, aadA-,* which were prevalent genes. The resistome composition displayed the highest co-occurrence to microbiome nodes for the sub-MIC 1/50 treatment with 23 ARGs, followed by the sub-MIC 1/100 treatment with 11 ARGs, and the sub-MIC 1/10 treatment with six ARGs. Aminoglycoside ARGs (*aph6-Id*, *rrsA*, *rrsB*) were present in all sub-MIC-exposed samples and correlated with Proteobacteria, as well as ARGs from other drug classes, except in control biofilms where these ARGs correlated only within the resistome composition (Supplementary Figure S11). The ARGs from the resistome nodes with the highest correlation to microbial nodes were *aph3”-Ia* and *blaOXA-2*. In the sub-MIC 1/10 treatment condition, *Aminobacter, Bosea, Caulobacter* and *Sphingorhabdus* genera showed a positive correlation to *sul1.*

This pattern indicates that Proteobacteria such as *Caulobacter, Bosea, Novosphingobium, Hydrogenophaga, Methylibium,* and more specifically *Variovorax* may act as potential reservoirs for aminoglycoside, β-lactam and sulfonamide resistance factors.

## 4. Discussion

### 4.1. Sub-MICs antibiotic affects the structure of riverine biofilm communities

Biofilm communities favor the maintenance and transmission of antibiotic resistance due to their diverse composition, close cell proximity, structural stability, and physical protection against chemical and physical stressors (Balcázar et al., 2015; Abe et al., 2020; Flores-Vargas et al., 2021; Matviichuk et al., 2022). In this study, heterogeneity between and within communities was observed, with up to a 54-μm range in biofilm thickness observed in samples from the same treatment (Figure 2). However, the effects of sub-MIC antibiotic treatments were most notable on biofilm communities developed under the sub-MIC 1/10 condition, where significantly thinner biofilms compared to other treatments (*p* < 0.05) were observed. Thinner biofilms have previously been noted to form in the presence of chemical stressors (Romero et al., 2018); therefore, thicker biofilms would seemingly proliferate under normal (non-stressed) growing-conditions. Thicker biofilms imply more diffusional distance within the matrix biomass for chemicals (i.e., antibiotics) to react with, thus initially providing more time (and reduced effective compound concentration) for microbial cells within the biofilm to adapt to stressor conditions. Furthermore, thicker biofilms imply greater protection provided by the volume of the EPS matrix, which is associated to increased cell density and closer proximity of cells (physical juxtapositioning), and consequently higher likelihood of cell–cell communication (quorum sensing) and genetic exchange which would facilitate HGT events (Dynes et al., 2009; Abe et al., 2020).

Carbohydrates are the structural skeleton of EPS matrix and can range from oligosaccharides through to polymers of up to 100 kD in size (Hanlon et al., 2006). Although polysaccharide composition in EPS of biofilms is highly heterogenous, most consist of a *N*-acetylglucosamine core (Flemming et al., 2022). The role of *N*-acetylglucosamine in EPS has mostly been studied in clinical monoculture biofilms such as *Pseudomonas aeruginosa* (Reichhardt et al., 2020), and is also known for being one of the major components of cell wall in bacteria (Gonzalez-Martinez et al., 2014). Result from our CLSM approach targeting glycoconjugate residues show a clear reduction of EPS polysaccharides, which could translate into lower signaling and hindered genetic exchange (Zhang et al., 2015). Since among biofilm elements, cyanobacteria and green algae exude large quantities of polysaccharides and amino-acids among other organic compounds (Battin et al., 2016; Delattre et al., 2016), it is reasonable to assume that the decrease of autotrophic organisms in biofilms treated with sub-MIC antibiotics, particularly at sub-MIC 1/10, had repercussions involving EPS accumulation (Figures 1, 3).

Riverine biofilms play a key role in river biogeochemical organic and inorganic cycles (Romaní et al., 2014) and EPS can be used as carbon source and energy reserves for bacterial heterotroph community and macrofauna (Pierre et al., 2012; Delattre et al., 2016), thus EPS reduction could hinder the further development of aquatic microorganisms. Additionally, EPS accumulation is crucial for adhesion to substrates (Fulaz et al., 2019), meaning that a reduction of EPS could contribute to earlier and/or higher frequency of detachment of biofilms, and further promote the spread of ARB and elements containing ARGs to and throughout the surrounding environment.

Biofilm formation in response to sub-MIC antibiotics remains contradictory even in single, clinically relevant bacterial isolates. For example, a commonly cited study demonstrates sub-MICs of aminoglycosides increased biofilm formation in *P. aeruginosa* and *E. coli* (Hoffman et al., 2005). However, a reduction in biofilm formation was reported for sub-MIC of β-lactams in *Staphylococcus* sp. (Cerca et al., 2005), macrolides in *Mycobacterium avium* (Carter et al., 2004), and mupirocin in *P. aeruginosa* (Horii et al., 2003). A more recent study documented how the presence of several antibiotics at sub-MIC levels affected *S. aureus* biofilm formation (Majidpour et al., 2017), where azithromycin and vancomycin increased biofilm formation, but linezolid, cefazolin, and clarithromycin inhibited it. A similar response was observed in *Staphylococcus epidermidis* biofilms where 0.5 MIC ciprofloxacin reduced biofilm thickness, but 0.5 and 0.25 MIC tigecycline increased biofilm growth, as well as the expression of *icaA*, *altE*, and *sigB* genes involved in polysaccharide intercellular adhesion (Szczuka et al., 2017). These studies are consistent with our microbiome-level results, given that sub-MIC antibiotic cocktail mix treatments containing ciprofloxacin in range between 5 to 50 μg/L decreased biofilm structural complexity. These findings demonstrate that effect on biofilm formation and its constituent members is likely antibiotic-dependent; thus, antibiotics affect diverse bacteria within biofilms differently. Indeed, the bioavailability and chemical state of antibiotics would also be presumed to influence the severity of response and/or impact on the biofilm, though these parameters are not within the scope of the current study.

In addition to chemical and physical stressors, biofilms are also exposed to biological stressors that include nutrient availability, protozoan predation and viral lysis (Andersson et al., 2018). Different protozoa have the potential to influence the proportion of bacterial species via selective or non-selective grazing, and have been shown to promote the occurrence of HGT events during short time frames (Sun et al., 2018). Protozoa were first detected in the fourth week of treatment exposure, with counts increasing slightly over the following weeks of the experiment, in alignment with the expected biofilm’s structural maturation throughout the eight-week period. As the biofilm matures, increased thickness and biomass would be expected to result in increased grazing pressure by protozoan organisms (Lawrence et al., 2009). Protozoa counts decreased in the presence of sub-MIC antibiotics, though we could not correlate the sub-MIC treatment to the protozoa composition or predation-interactions. It is possible that the observed reduction of protozoa organisms in biofilms under sub-MIC exposure was due to the limited availability of autotrophic bacteria recorded under sub-MIC antibiotic conditions, indicating an indirect effect through trophic interactions (Figure 1; Supplementary Figure S1). It has been previously reported that high protozoan predation on bacteria was associated with persistence of *Mycobacterium* and *Rickettsia* spp. (Andersson et al., 2018). *Mycobacterium* spp. were prevalent taxa throughout the biofilm communities of this study; thus, it is possible that predatory protozoa in these samples would be associated with, and contribute to the maintenance of, *Mycobacterium* spp.

Ultimately, reduced EPS results in an increase in the proportion of unprotected bacterial biomass but a reduction in protozoan and cyanobacteria (autotrophic bacteria). Loss of diversity and decreased protection from the EPS matrix signify a broader ecological impact of sub-MIC antibiotic exposure that could further accelerate the acquisition or expression of resistance functions of bacteria species within biofilm communities.

### 4.2. Microbiome composition shifts due to sub-MIC antibiotic exposure

Microbial composition analysis revealed that Proteobacteria species were favored by sub-MICs antibiotic presence, and overall, a considerable shift in bacterial community composition was observed at sub-MIC 1/10. Relative abundance profiles in this study (Figure 4) were in accordance with previous taxonomic results noted for South Saskatchewan River biofilms by Lawrence et al. (2020), where the dominant phyla (Proteobacteria, Actinobacteria and Cyanobacteria) comprised 63% of total bacterial community membership. Proteobacteria, particularly *Betaproteobacteria*, are implicated in more readily attaching to surfaces during initial biofilm development (Luo et al., 2020). Proteobacteria predominance throughout treated biofilm communities also signals their adaptability to low or sub-MICs antibiotics (Manaia, 2017) supporting the idea that species within this phylum possess antibiotic resistance mechanisms or potential for acquired resistance.

Additionally, river biofilms influenced by nearby run-off from WWTPs also showed Proteobacteria to be the dominant phyla (Guo et al., 2020; Matviichuk et al., 2022). Our results are in agreement with Zou et al. (2018) where oxytetracycline residues selected for Proteobacteria abundance. In contrast, Subirats et al. (2018) did not detect significant variations in bacterial abundance of biofilms treated with ciprofloxacin, erythromycin, sulfamethoxazole, diclofenac, and methylparaben, even though these antibiotics are known to inhibit bacterial growth at sub-MIC levels. Notably, that experimental design comprised 28 days, instead of the 56-day period used for this study. Thus, it is possible in Subirats et al. that the EPS matrix provided protection during early sub-MICs antibiotic exposure. However, the prolonged period of antibiotic exposure in our study might indicate the scale at which ecological impacts may be observed due to resultant shifts in microbial community composition.

nMDS analysis demonstrated that sub-MIC 1/10 and sub-MIC 1/50 treatments most profoundly shaped microbiome composition at the phyla and genus level. For example, *Brevundimonas* and *Methylorubrum* (Proteobacteria) as well as *Flavobacterium* (Bacteoridetes) relative abundance displayed significant correlation to biofilms grown in sub-MIC 1/50 antibiotic treatment; interestingly the two latter genera also demonstrated co-occurrence to *Variovorax* in the network analysis (Figure 10). *Nitrospira* (Nitrospirota phylum) revealed to be strongly associated with control biofilms (Figure 6) and showed co-occurred with several ARGs and genera in sub-MIC 1/50 treated biofilms. The prevalence of *Mycobacterium* spp. despite reduced abundance (up to 50%) in the presence of sub-MIC antibiotics (relative to control biofilms) presumably indicates that Actinobacteria were sensitive to the effects of antibiotics at these concentrations, or that antibiotics hindered the development of these genera.

Calculation and interpretation of α-diversity and species richness proved to be very dependent on diversity indices, taxonomic level, and databases (Supplementary Figures S4, S5). Bacterial α-diversity data of biofilms in the South Saskatchewan River is limited, however results from CosmosID and Kraken databases demonstrated that biofilms in this study were similar in diversity and community distribution to previous microcosm characterizations (Romaní et al., 2014). Furthermore, diversity distribution was similar between the sub-MIC 1/10 and sub-MIC 1/50 samples, which were generally less diverse than controls. This is not unexpected as it has been previously recorded that bacterial richness, diversity and composition are disturbed by the presence of antibiotics such as ciprofloxacin (Gonzalez-Martinez et al., 2014) and oxytetracycline (Zhang et al., 2013).

In line with this observation, it is frequently concluded that a decrease in diversity is an indicator of altered microbial taxonomy and by extension, functional environmental health functions and services. However, this data cautions against using α- or β-diversity as a sole metric or predictor of ecological impact or health in face of environmental stresses, given the dependence on database health, data normalizations and granularity of taxa level (Shade, 2017).

### 4.3. Comparisons of microbial reference databases

To our knowledge, this is the first study to evaluate different commonly used resistome and taxonomic databases for environmental aquatic microbiomes; and thus can serve to provide guidance for future study considerations and best practices. CosmosID, Kraken and MetaPhlAn are commonly-used analysis pipelines that were all separately applied in this study to profile taxa of biofilm communities (Couto et al., 2018). These analysis tools and pipelines notably influenced observations, though consensus data of bacterial abundance was achieved. These differences in data outputs are a result of the underlying databases used for alignment. Specifically, Kraken uses genomes based on the NCBI RefSeq database consisting of over 128,299 organisms (O’Leary et al., 2016); MetaPhlAn compares sequences against the CHOCOPhlAn_201901 database v.4.0 (Segata et al., 2012), containing over 771,500 metagenomic assembled genomes; and CosmosID aligns against the GenBook database (150,000 microbial genomes and 5,000 viral species) using k-mer-based alignment (Yan et al., 2019; Zaouri et al., 2020). In the present study, MetaPhlAn was less able to identify sequences to the species level (20 of 59 species were categorized as unclassified), whereas Kraken assigned the most sequences to species level.

Similar to taxonomic analysis, three different commonly used resistome databases were evaluated for sensitivity and accuracy of ARG annotation of metagenomes: CosmosID (5,500 ARGs), CARD (1,600 manually-curated ARG sequences) and ResFinder (3,000 ARG). Again, the selection of database directly affected final observations; however, all three analysis streams displayed the trend of sub-MIC antibiotics selecting for an increase in ARG abundance within biofilms. Correlation analysis revealed that CARD and ResFinder databases were most similar in their output and performance (Supplementary Figure S7B).

Despite the known challenges of comparing metagenomic data between studies (Couto et al., 2018), the dependence of data interpretation and conclusions on database quality, environmental metagenomics studies frequently present data produced from a favored tool or database. By performing analysis using one method and failing to compare the output against other available tools, particularly in the field of environmental AMR, researchers’ risk either under- or over-interpreting their results. Here we conclude that the CARD database and associated tools are currently the most reliable resistome database due to its high level of curation and maintenance, and that the CosmosID database is a reliable source for taxonomic profiling of aquatic environmental microbiomes, especially if the study seeks to identify Gram-negative or pathogenic bacteria within samples (Couto et al., 2018).

### 4.4. Resistome composition enhanced after sub-MIC antibiotic exposure

Resistome profiling of riverine biofilms developed under a sub-MICs antibiotic cocktail mix of ciprofloxacin, oxytetracycline and streptomycin revealed an abundance prevalence and dominance for aminoglycoside ARGs (Supplementary Figure S3). Resistome distribution classified by drug resistance and mechanism was consistently similar across the three resistome pipelines, with sub-MICs antibiotic treated biofilms yielding greater number of resistome elements in comparison to control biofilms (Supplementary Figure S6). These findings are consistent with resistome abundance observed in biofilms influenced by hospital wastewater (Petrovich et al., 2020) and WWTP discharge (Matviichuk et al., 2022). Microbiome composition aligned with resistome results in that the highest richness of taxa/genes were registered for the sub-MIC 1/50 treatment in comparison to control biofilms. Overall, *aph6-Id*, *aph3”-Ib*, aadA2 *tetC*, *tetG, sul1*, and *sul2* were highly abundant and/or prevalent across all biofilms under sub-MICs exposure (Supplementary Table S3).

It has been previously reported that aminoglycoside resistance genes co-select with other ARGs such as those encoding β-lactamases and are often located within different mobile genetic elements (MGE) such as integrons and plasmids (Beceiro et al., 2013). Since ARGs are often co-located with pathogen virulence factors in biofilm organisms, we also screened for the occurrence of virulence genes. Virulence refers not only the ability of bacteria to cause disease in the host (i.e., degree of pathogenicity) but also the ability of an organism to infiltrate and colonize a host (Schroeder et al., 2017). *TpnA* transposase and *sul1* sulfonamide genes were notably prevalent across all treatments including control biofilms according to the VFDB database (Supplementary Figure S8). *TpnA* has been reported to commonly occur in drinking water systems (Brumfield et al., 2020), whereas *sul1* and *sul2* have been recorded to be prevalent ARGs in influent and effluent riverine biofilms (Auguet et al., 2017).

Interestingly, relative abundance of *intI1,* a class I integron gene usually located in MGE such as transposons and plasmids (Gillings et al., 2015) and linked to several resistance genes including *sul1* (Subirats et al., 2018; Cheng et al., 2020), was enhanced in all sub-MICs antibiotic treated samples. The *sul1* ARG is of significant clinical relevance (Petrovich et al., 2020) and its abundance has been found to be greater in extracellular DNA than intracellular DNA in estuarine biofilms (Guo et al., 2018), supporting the idea that mobilization of this gene plays an important role in resistance transmission. The prevalence of *intI1, sul1* and *tpnA* genes across biofilms under sub-MICs antibiotic exposure might be result of their mobilization across bacteria, particularly Proteobacteria species, as HGT dissemination events are more common between phylogenetically related bacteria (Subirats et al., 2018). Given this increase in suspected HGT events and the fact that constant sub-MICs exposure of antibiotics can select for tolerance mutations (Schuster et al., 2022) it is plausible to assume that presence of both virulence and ARGs would result in multiple drug resistance within constituent microorganism (Schroeder et al., 2017). However, there is still not a clear understanding of the co-selection relationship(s) between virulence and resistance factors, nor virulence and antibiotic concentrations (Beceiro et al., 2013; Matviichuk et al., 2022). Therefore, tracking AMR in environmental microbiomes is an essential step required to anticipate the spread and risk of ARGs/ARB transmission.

Environmental concentrations of ciprofloxacin in European WWTP effluent released into surface freshwater were higher than estimated and found to promote resistance in 5% of bacterial species in contact with the antibiotic (Emara et al., 2023). The same study reported a similar pattern for tetracycline, trimethoprim, ofloxacin, and norfloxacin. Recently, ciprofloxacin (among other antimicrobials) has been measured in the range of 542–32,800 ng/L in effluents close to two Canadian pharmaceutical manufacturing facilities (Kleywegt et al., 2019). It has also been determined in France that river biofilms exposed to WWTP discharge with oxytetracycline concentrations ≥ to 108 μg/L represented an environmental risk for aquatic bacteria (Matviichuk et al., 2022). These *in-situ* concentrations are within the range used in our present study (Supplementary Table S1), with values covering 1/10, 1/50, and 1/100 of MIC and ranging from 5,000 to 50,000 ng/L (below MICs of 500,000 ng/L), supporting the notion that global freshwater microbial communities are currently exposed to sub-MICs of antibiotics, with potential functional and structural impacts such as those observed in this study.

### 4.5. Broader microbial functional impacts

Significant changes in metabolic pathways found within the whole biofilm community under sub-MICs antibiotic exposure were also observed. In general, genes belonging to metabolic pathways related to biosynthesis processes were differentially abundant in treatment biofilms. For example, two peptidoglycan pathways were similarly abundant in each of the three sub-MIC antibiotic treatments; suggesting that even at the lowest concentration (5–5,120 μg/L) antibiotic presence can increase peptidoglycan biosynthesis. This upregulation of cell wall biosynthesis is likely in response to sub-MICs antibiotics exerting selective pressure on microbial communities, and in particular, the abundant group of Proteobacteria (Gram-negative bacteria) present. β-lactam antibiotics can interfere with the bacterial cell wall biosynthesis by inactivation of penicillin-binding proteins (PBPs), enzymes requisite for synthesis of the peptidoglycan layer (Pazda et al., 2019). β-lactam resistance mechanisms against Gram-negative bacteria mostly consist in β-lactamases, followed by permeability alterations and extrusion by efflux pumps (Beceiro et al., 2013); *blaOXA*-10 and *blaOXA*-24 have further been implicated in virulence properties by changing peptidoglycan composition (Fernández et al., 2012). Results from our functional prediction demonstrate that sub-MICs antibiotics enhanced the abundance of peptidoglycan-related metabolism, with a similar pattern observed for the abundance of *blaOXA*-1 and *blaOXA*-2 genes.

Functional metagenomic analysis also revealed shifts in gene abundance related to amino acid biosynthesis pathways at sub-MIC 1/10 and 1/50 conditions, including those responsible for synthesis of arginine, lysine, histidine, and isoleucine (Figure 9). Genes associated with lysine biosynthesis have been shown to be required in bacteria for protein synthesis, cell wall biosynthesis and generation of diaminopimelate (DAP), the latter being a key cell wall component in many Gram-negative bacteria (Grundy et al., 2003). Nucleotide and nucleoside biosynthesis genes consistently showed reduced (negative fold-change) gene abundance in treated samples relative to control samples. These results indicate potential disruptions in bacterial growth, since genes associated with these pathways are key in synthesizing precursor metabolites for central anabolic processes (Caspi et al., 2018). Considering that antibiotic resistance often causes fitness costs (Andersson and Hughes, 2012), shifts in genes associated with nucleoside and nucleotide pathways and overall growth rates could be related to the increased replication demands from more abundant MGE such as plasmids or integrons.

Interestingly, no other metabolic pathway genes associated with stress response or resistance displayed significant differential abundance in response to sub-MICs antibiotic exposure. In addition, no significant variations in carbon-related gene abundance were observed. This was not surprising given that carbon biosynthesis would largely result from autotrophic and/or microalgae organisms (non-prokaryotic) processes which were excluded from bacterial gene abundance screen regarding metabolic pathway analyses. Overall, sub-MIC antibiotic exposure seemingly influences cell wall biosynthesis and processes related to bacterial growth regulation. As biofilms are key participants in biogeochemical organic and inorganic cycles, including primary productivity, alterations to biofilm growth dynamics can potentially lead to downstream impacts on these environmental functions.

### 4.6. Microbiome and resistome linkages post-antibiotic exposure

Network analysis patterns from biofilm communities at different sub-MIC antibiotic conditions demonstrated that the overall resistome composition correlated to Proteobacteria genera.

Proteobacteria genera such as *Bradyrhizobium*, *Methylibium, Methylorubrum*, *Novosphingobium, Sphingobium* and *Variovorax* showed the highest correlations (number of edges) to the resistome (Figure 10; Supplementary Figure S11) suggesting that the presence of these genera strongly contribute to shaping the microbial composition and that they may play important ecological roles in riverine biofilms.

With respect to ARGs composition, aminoglycosides (*aadA3, aph3” Ib, aph6-Id, rrsA*), β-lactam (*blaOXA-2*, *blaOXA-21*), and sulfonamide (*sul1*) were the most prevalent across biofilms under sub-MICs antibiotic exposure (Figure 10). The *aph6-Id* gene displayed co-occurrence to *aph3”-Ib* in all sub-MICs treatments, and correlation with other ARGs increased with the concentration of sub-MICs antibiotics. Indeed, the co-occurrence of Proteobacteria, particularly *Variovorax*, suggests that this bacterial genera could act as potential host of aminoglycosides, β-lactams and sulfonamides and trimethoprim ARGs.

Moreover, Proteobacteria taxa, *Mycobacterium* and *Mycobacteroides* (Actinobacteria) showed correlation to *blaOXA-2, sul1*, and *tetG* genes. Occurrence of the *sul1* gene was negatively correlated to *Nitrospira,* showing different observations from that of Matviichuk et al. (2022) who reported that *sul1* was correlated with Bacteroidetes and Nitrospirae phyla from samples upstream of a WWTP, but with no correlation in downstream biofilms. Though co-occurrence patterns between resistome and microbiome across the different sub-MICs antibiotic conditions were observed, this approach and dataset is limited in its ability to ascribe with certainty the abundance of resistance genes to specific bacterial taxa (Supplementary Figure S11). However, the totality of our results suggests that predominance of Proteobacteria could serve as an indicator of AMR risk and aid in defining strategies for aquatic environmental surveillance efforts, given that the abundance of this taxa group is strongly correlated with ARG abundance.

## 5. Conclusion

This study demonstrated that the combined presence of sub-MICs oxytetracycline, ciprofloxacin and streptomycin in riverine biofilm communities decreased microbial diversity and selected for Proteobacteria genera including *Methylorubrum* and *Variovorax*, particularly at the sub-MIC 1/10 antibiotic level. In contrast, the relative abundance and richness of resistome composition increased at the sub-MIC 1/50 antibiotic level, suggesting greater opportunities for HGT events and transfer of MGE occurred under this condition. At sub-MIC 1/50 antibiotic conditions, exposure did not affect EPS matrix thickness as significantly as observed for the 1/10 treatment level, thereby potentially enabling enhanced cell–cell communication (quorum sensing) between biofilm microorganisms. Accordingly, there are a range of biofilm impacts and lifestyle trade-offs, with respect to structural stability and genetic diversity across the range of sub-MICs concentrations examined here. Our results have important implications for the overall functional capacity, ecosystem services, and broader ecological roles that sub-MICs antibiotic impacted biofilms play in aquatic ecosystems. Altogether, these observations confirm that the presence of environmentally relevant concentrations of antibiotics promote and accelerate the acquisition of resistance in aquatic microbial communities.

## Data availability statement

The datasets presented in this study can be found in online repositories. The names of the repository/repositories and accession number(s) can be found at: https://www.ncbi.nlm.nih.gov/, BioProject ID: PRJNA944808.

## Author contributions

GF-V wrote the manuscript and performed experiments and data analysis. JB wrote and reviewed manuscript and designed and supervised research. DK wrote and reviewed manuscript and designed and supervised research. All authors contributed to the article and approved the submitted version.

## Funding

Financial support for this research was provided by Environment and Climate Change Canada and the University of Saskatchewan. Also, support of the Natural Sciences and Engineering Research Council of Canada (NSERC; grant to DK) is acknowledged. This work was also supported by Nous remercions le Conseil de Recherches en Sciences Naturelles et en Génie du Canada (CRSNG) de son soutien.

## Conflict of interest

The authors declare that the research was conducted in the absence of any financial relationship that could be construed as potential conflict of interest.

## Publisher’s note

All claims expressed in this article are solely those of the authors and do not necessarily represent those of their affiliated organizations, or those of the publisher, the editors and the reviewers. Any product that may be evaluated in this article, or claim that may be made by its manufacturer, is not guaranteed or endorsed by the publisher.

## Footnotes

^1^https://github.com/lh3/seqtk

^2^http://huttenhower.sph.harvard.edu/humann
