# Supplementary material for: Sub-MIC antibiotics influence the microbiome, resistome and structure of riverine biofilm communities
Source: Front Microbiol. 2023 Aug 1;14:1194952. doi: 10.3389/fmicb.2023.1194952 (PMC10427767; doi:10.3389/fmicb.2023.1194952)
Supplement: Supplementary file 3 [file Data_Sheet_1.docx]

Supplementary Material

**Sub-MIC antibiotics influence the microbiome, resistome and structure of riverine biofilm communities**

**Gabriela Flores-Vargas ^1^, Darren R. Korber ^1^, Jordyn Bergsveinson ^2*^**

^1^ Food and Bioproduct Sciences, University of Saskatchewan, Saskatoon, SK, S7N 5A8, Canada

^2^ Environment and Climate Change Canada, 11 Innovation Blvd., Saskatoon, SK, S7N 3H5, Canada

* **Corresponding author**:

Dr. Jordyn Bergsveinson

[jordyn.broadbent@ec.gc.ca](mailto:jordyn.broadbent@ec.gc.ca)

# **Supplementary Figures**


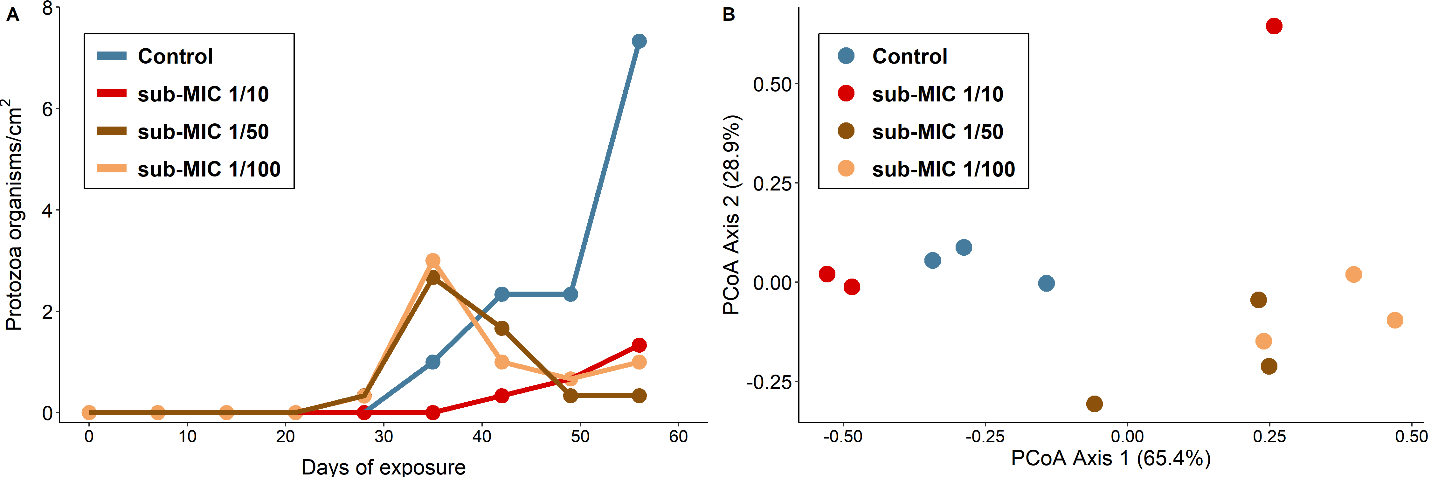


**Figure S1**. Protozoa composition in biofilm communities under sub-MIC antibiotic exposure. A) Protozoa enumeration and B) PCoA protozoa ordination based on Bray-Curtis distances (*n* = 3)

**
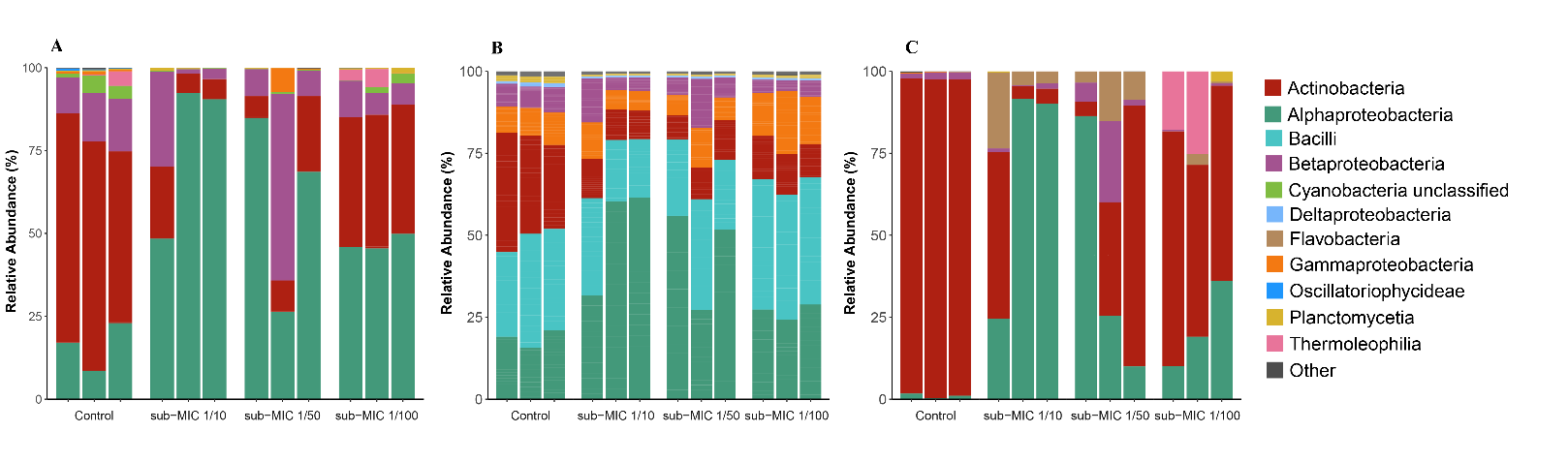
**

**Figure S2**. Comparison of taxonomic classification tools. Relative abundance of A) CosmosID, B) Kraken, and C) MetaPhlAn bacterial profiles at the class level.


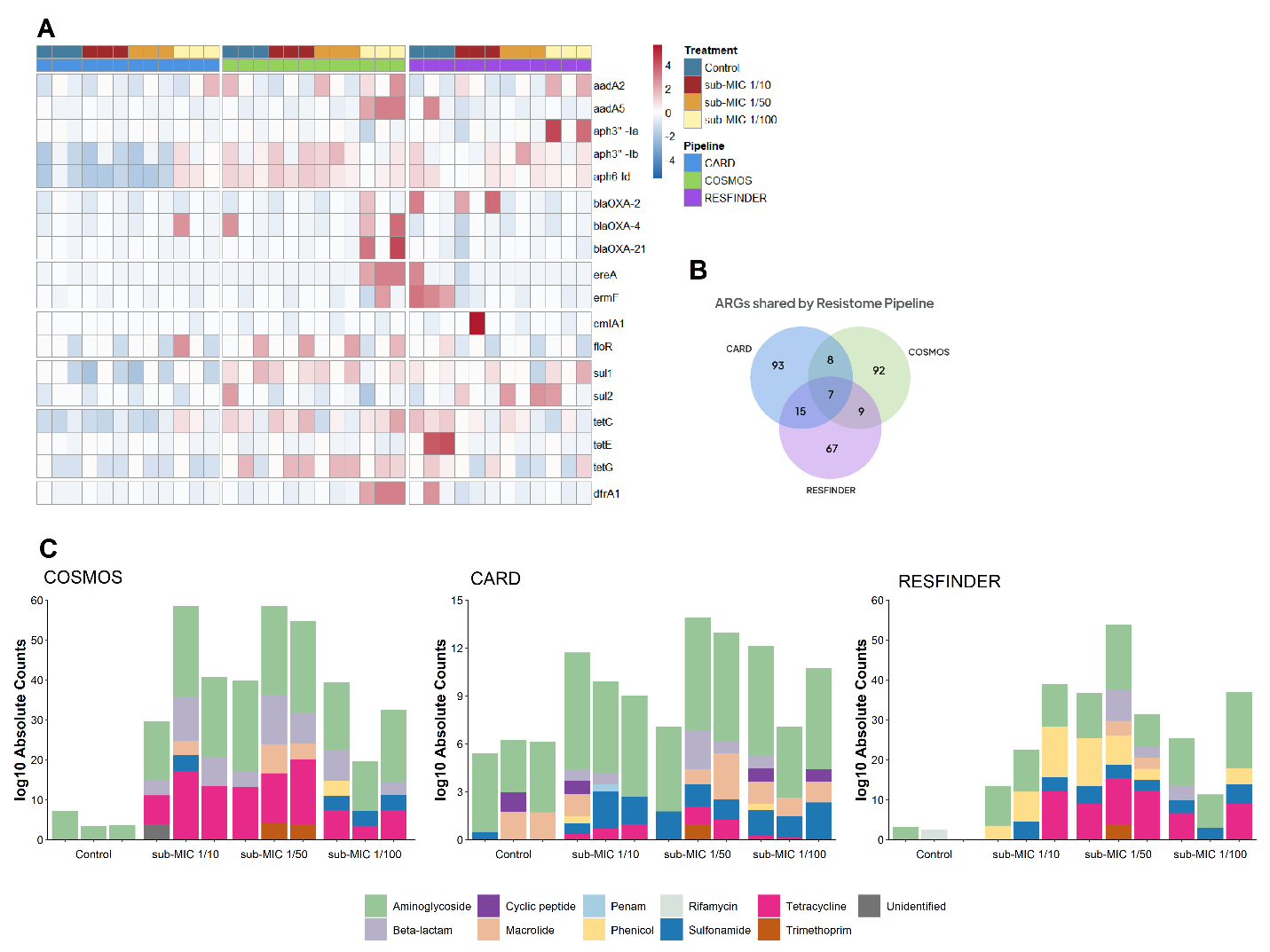


**Figure S3**. Absolute counts (abundance) of the resistomes grouped by resistance to drug class across biofilm communities under sub-MIC antibiotic exposure. Absolute Counts indicates the normalized read counts annotated by ­the different databases.

**
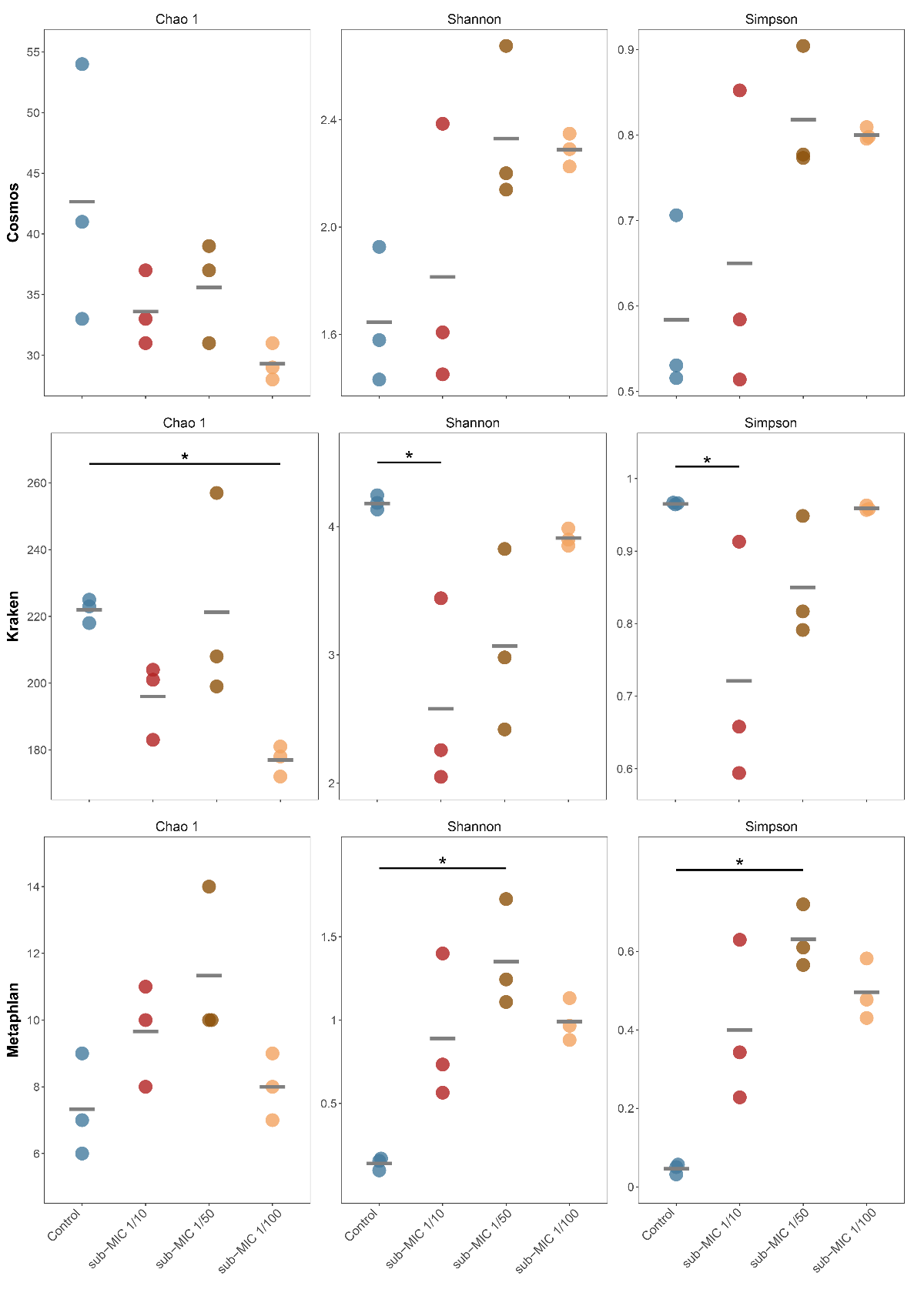
**

**Figure S4**. α diversity indices of bacterial profiles at the Genus taxa level identified though different taxonomic classification tools (CosmosID, Kraken and MetaPhlAn). Grey horizontal lines indicates mean values, and asterisks indicate significant differences (*p* < 0.05) between pairs connected by black horizontal lines from Kruskal-Wallis and post-Dunn test with *p*-value adjusted using the Benjamini-Hochberg method.


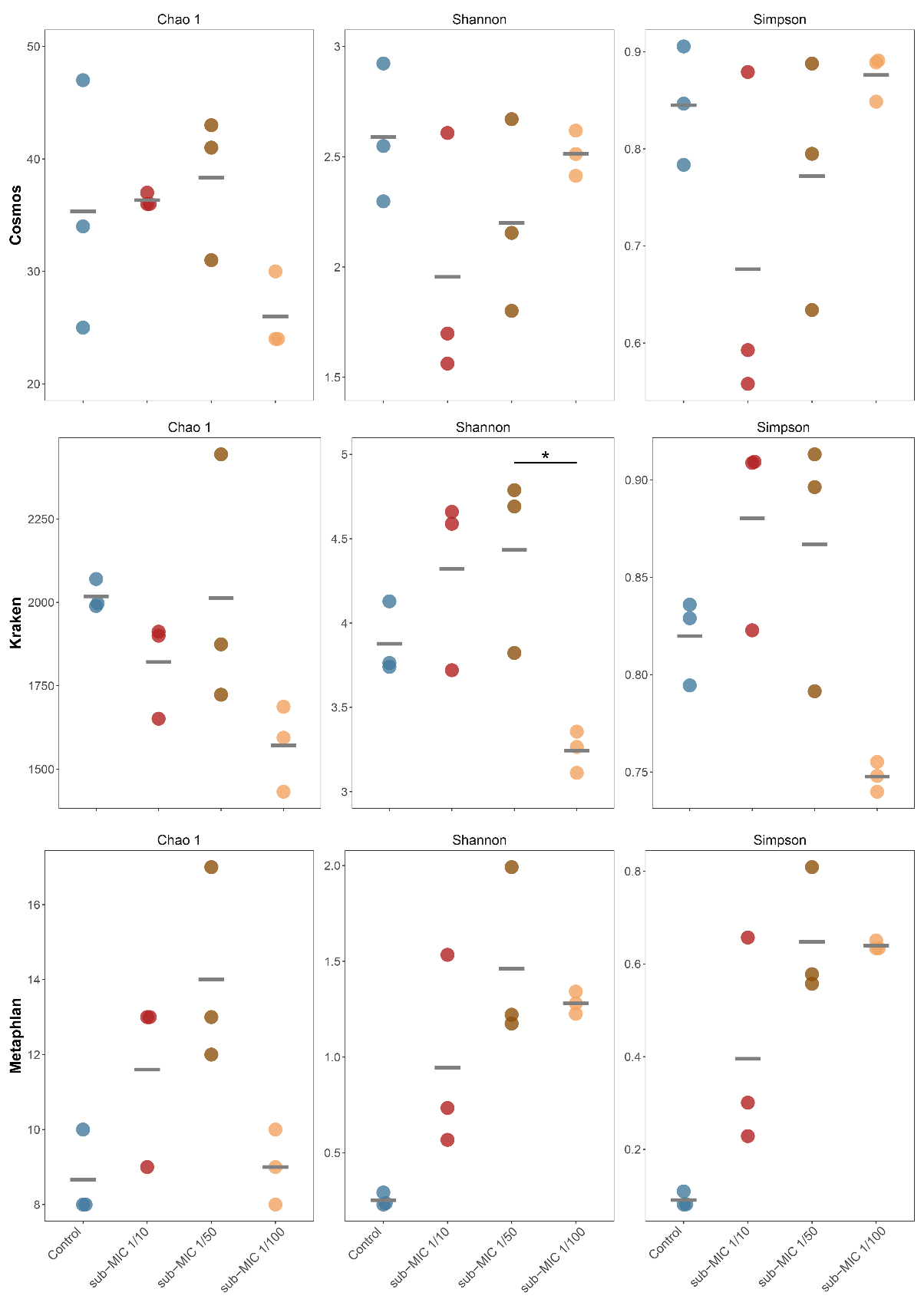


**Figure S5**. α diversity indices of bacterial profile at Species taxa level identified though different taxonomic classification tools (CosmosID, Kraken and MetaPhlAn). Grey horizontal line indicates mean values, and asterisk indicates significant differences (*p* < 0.05) between pairs connected by the black horizontal line from Kruskal-Wallis and post-Dunn test with *p*-value adjusted using the Benjamini-Hochberg method.

**
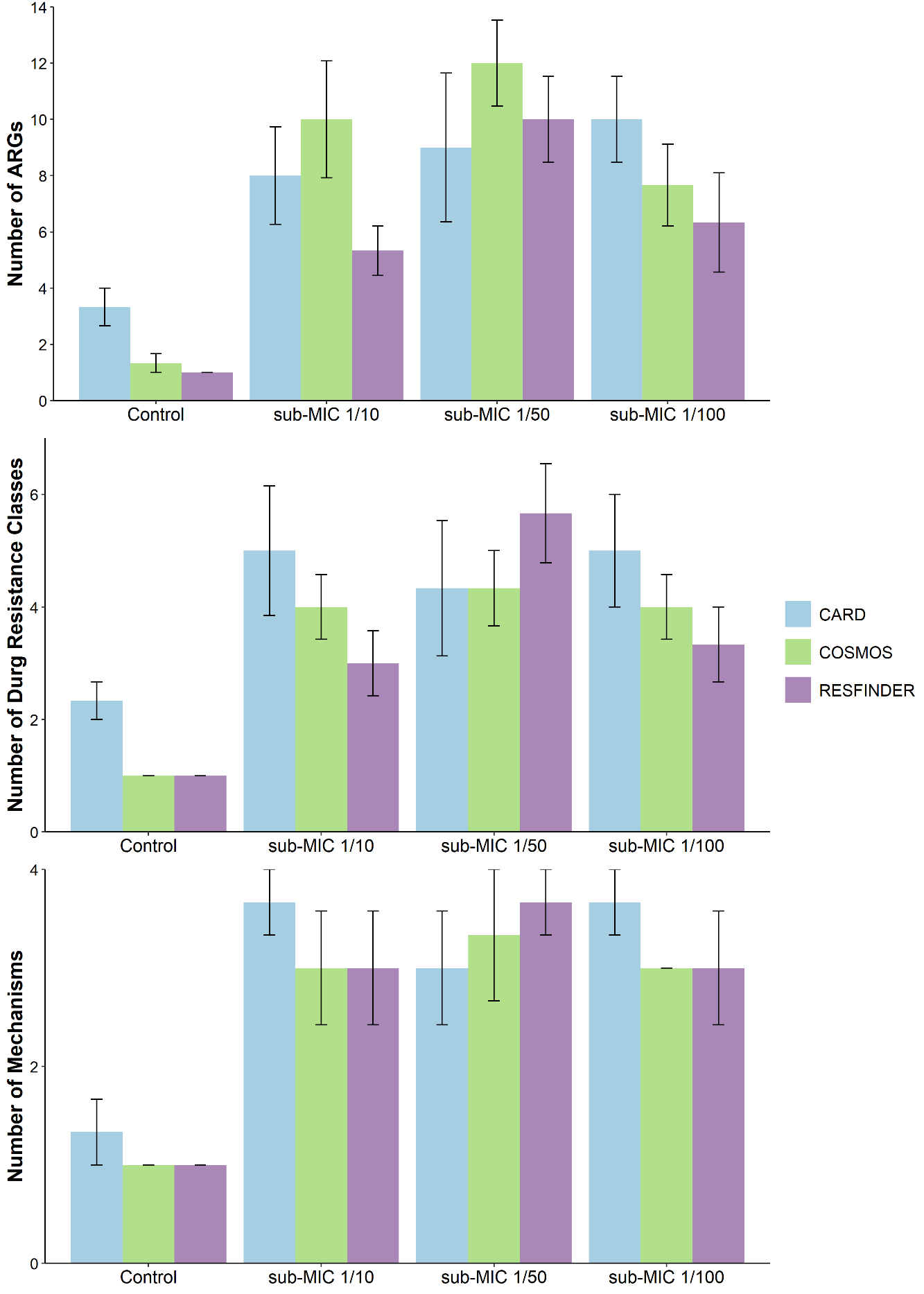
**

**Figure S6**. Observed resistome richness classified by the number of ARGs, drug classes and mechanisms of resistance across the three analyzed pipelines (CARD, CosmoID, ResFinder).


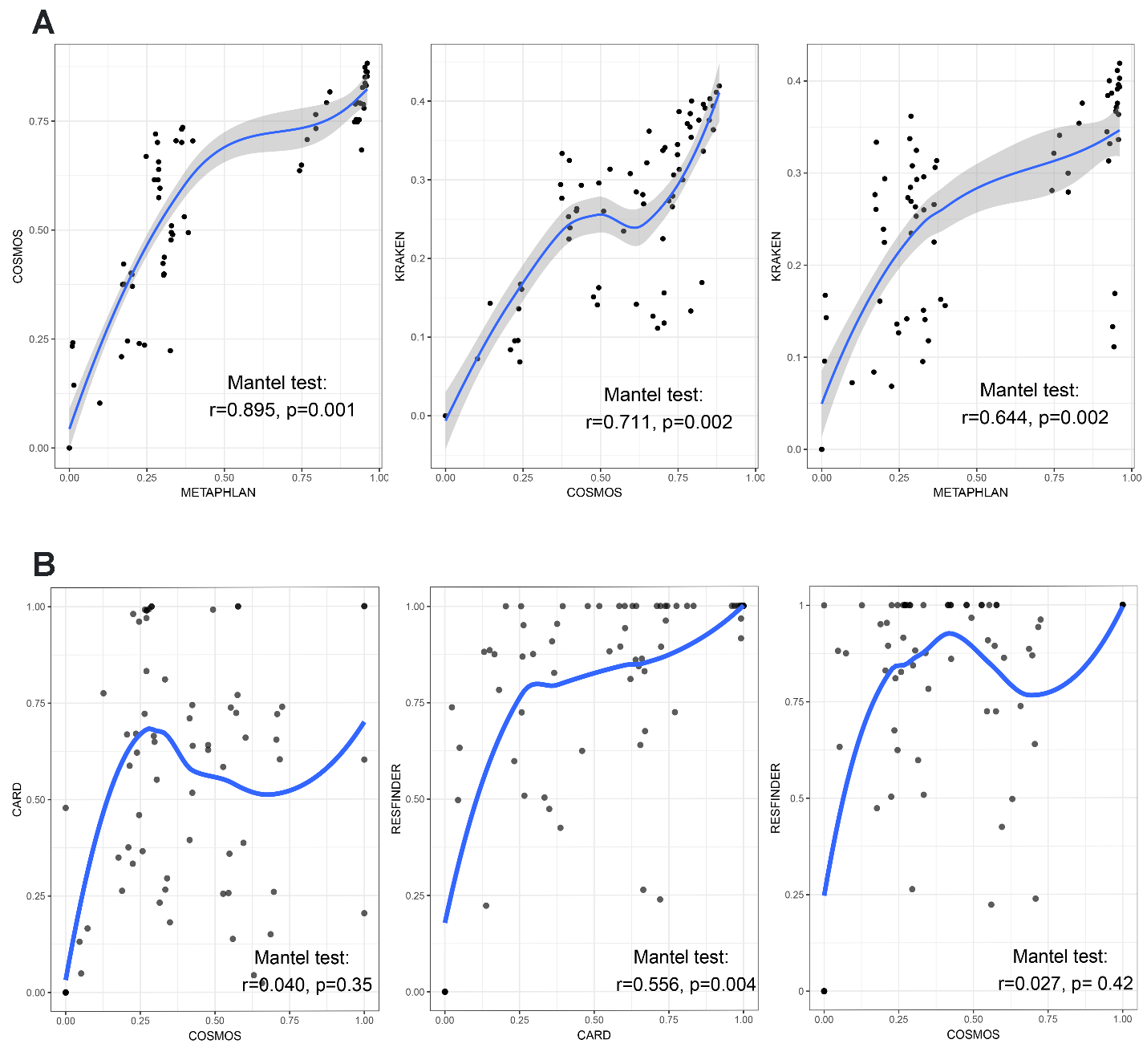


**Figure S7**. Comparison of A) microbiome (CosmosID, Kraken and MetaPhlAn) and B) resistome (CARD, CosmosID and ResFinder) databases on the number of identified OTU and ARGs, respectively (r= Spearman’s correlation coefficient; P= *p*-value).


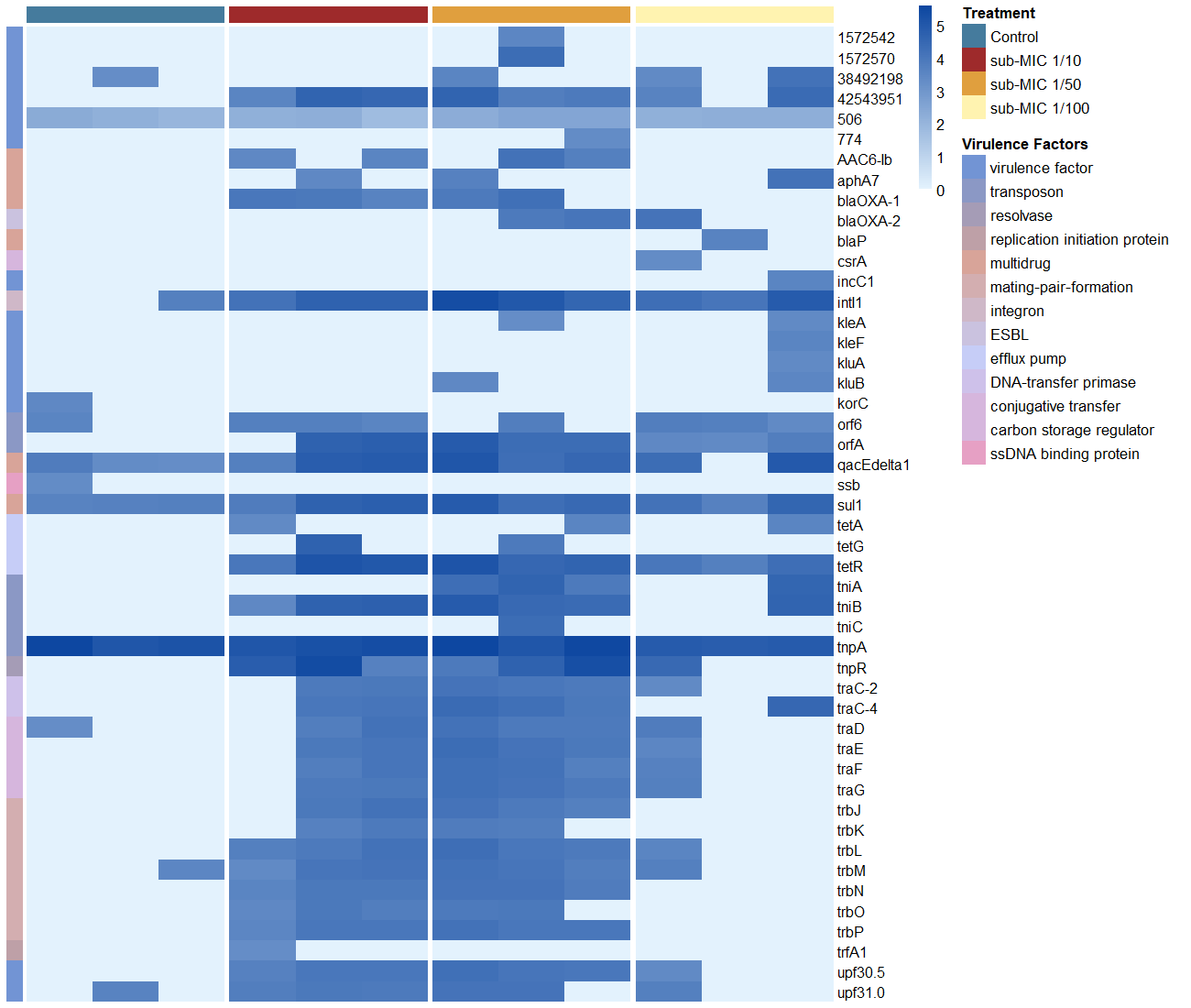


**Figure S8.** Virulence genes identified across biofilm communities under sub-MIC antibiotic exposure. Legend denotates absolute log_10_ abundance.

**
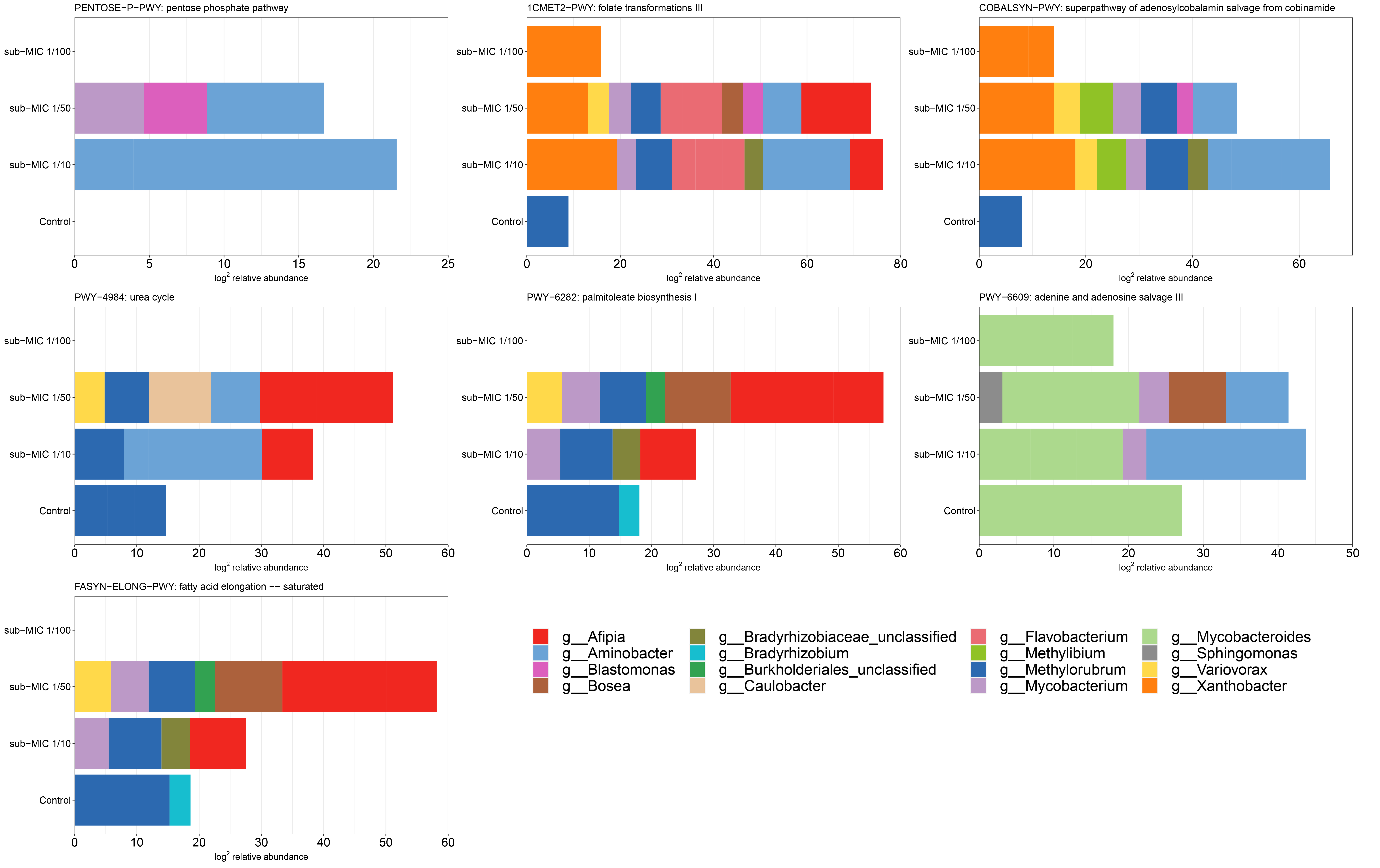
**

**Figure S9**. Relative abundance of functional pathways genes with most notorious differences across sub-MIC antibiotic treatments annotated with HUMAnN analysis using the MetaCyc database. X-axis represents normalized RPK units of each gene associated to the pathway abundance file. Each plot represents an individual metabolic pathway. Each bar represents a treatment (*n* = 3) and stratification within each bar represents bacterial species contribution at the genus level. Unclassified contributions were removed for visualization.


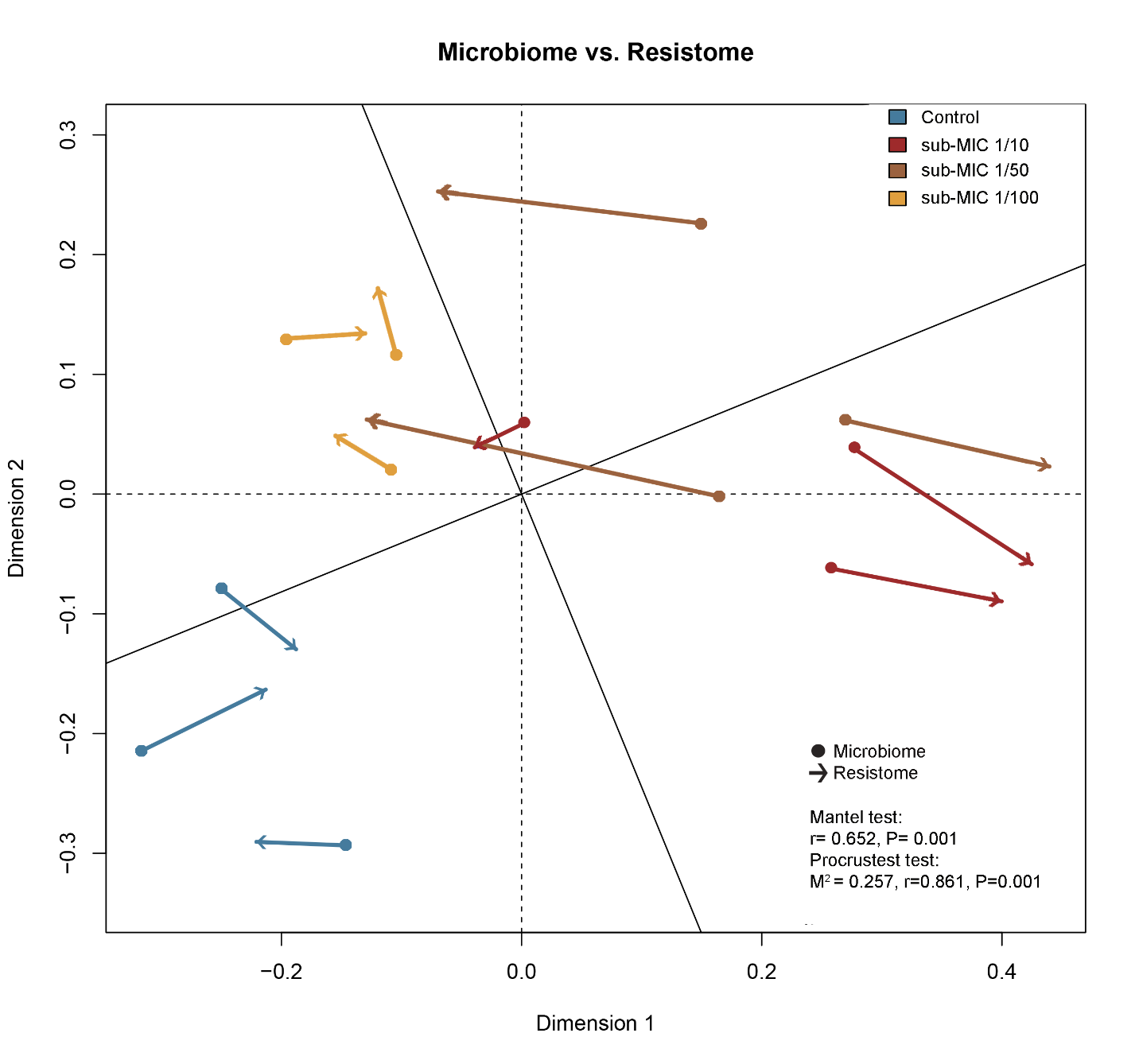


**Figure S10.** Procrustes analysis based on nMDS ordination showing correlation between abundance of bacterial composition at the genus level and resistome composition by ARGs in riverine biofilm communities under sub-MIC antibiotic exposure (r= Spearman’s correlation coefficient; P= *p*-value <0.001; M^2^= sum of squares).


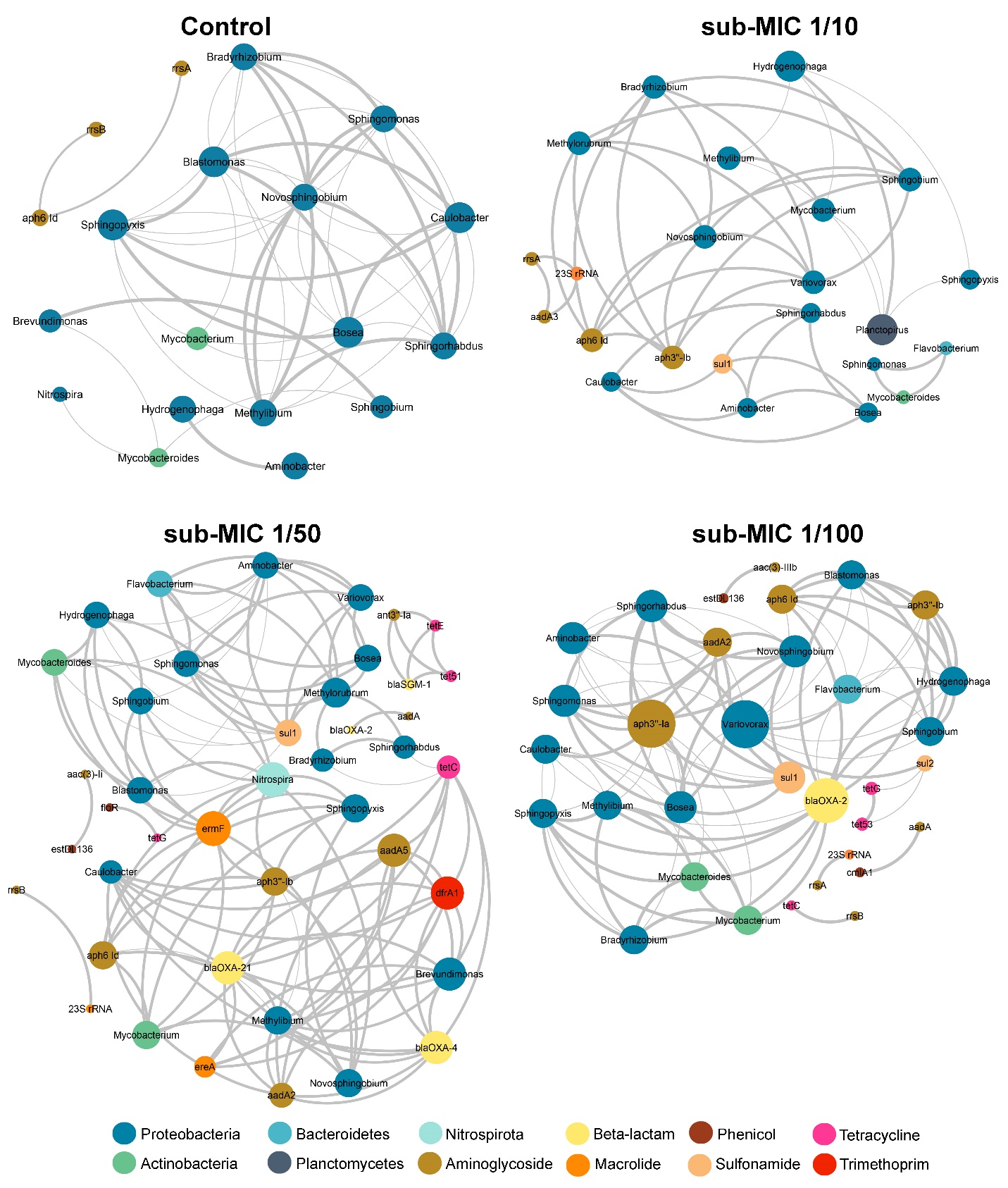


**Figure S11**. Network analysis showing correlation patterns between the microbiome and resistome at each of the sub-MIC antibiotics treatments. Edges (lines) connecting nodes mean a strong correlation (Spearman’s rho > 0.75, *p* <0.05), negative indicated in red and positive in grey. The size of each node is proportional to the number of connections (degree) (*n*=3).

# **Supplementary Tables**

**Table S1**. sub-MIC antibiotic cocktail mix for each treatment.

| **Antibiotic** | **MIC *** | **sub-MIC 1/10** | **sub-MIC 1/50** | **sub-MIC 1/100** |
| --- | --- | --- | --- | --- |
| Ciprofloxacin | 0.5 mg/L | 50 μg/L | 10 μg/L | 5 µg/L |
| Oxytetracycline | 125 mg/L | 12,500 µg/L | 2500 µg/L | 1250 µg/L |
| Streptomycin | 512 mg/L | 51,200 µg/L | 10,240 µg/L | 5120 µg/L |

* MIC defined for community based on extracted values from EUCAST and CLSI database.

**Table S2.** Number of raw, assembled and annotated sequences per sample.

**[ for complete list please see Excel file: “Reads_Report.xlsx” ]**

Table S3. Occurrence of ARGs detected at all 3 pipelines in biofilm communities under sub-MICs antibiotics exposure.

| **ARG** | **Drug Class** | **Mechanism** | **Control** | **sub-MIC 1/10** | **sub-MIC 1/50** | **sub-MIC 1/100** |
| --- | --- | --- | --- | --- | --- | --- |
| aadA5 | Aminoglycoside | Antibiotic inactivation | - | - | + | - |
| aph(3'')-Ib | Aminoglycoside | Antibiotic inactivation | - | + | + | + |
| aph6 Id | Aminoglycoside | Antibiotic inactivation | + | + | + | + |
| dfrA1 | Trimethoprim | Antibiotic target replacement | - | - | + | - |
| ereA | Macrolide | Antibiotic inactivation | - | - | + | - |
| ermF | Macrolide | Antibiotic target alteration | - | - | + | - |
| tetC | Tetracycline | Efflux pump | - | + | + | + |

**Table S4.** List of identified ARGs in each of the 3 resistome databases.

**[ for complete list please see Excel file: “ARG_Report.xlsx” ]**

**Table S5**. Network properties on co-occurrence patterns between resistome (23 ARGs) and microbiome (20 genera) in riverine biofilm communities under sub-MIC exposure

| **Topological properties** | **Numbers** |
| --- | --- |
| Nodes | 29 |
| Edges | 35 |
| Diameter | 5 |
| Modularity | 0.766 |
| Average degree | 0.036 |
| Average path length | 2.10 |

**Table S6**. Network properties on co-occurrence patterns between resistome (ARGs) and microbiome (genus taxa level) in riverine biofilm communities at each of the sub-MIC treatments exposure (*n* =3).

| **Topological properties** | **Control** | **sub-MIC 1/10** | **sub-MIC 1/50** | **sub-MIC 1/100** |
| --- | --- | --- | --- | --- |
| Nodes | 19 | 23 | 42 | 33 |
| Edges | 47 | 45 | 120 | 96 |
| Diameter | 4 | 2 | 3 | 5 |
| Modularity | 0.64 | 5.488 | 6.191 | 1.579 |
| Average degree | 2.868 | 7.652 | 7.808 | 5.818 |
| Average path length | 1.585 | 1.38 | 1.739 | 2.0526 |
